# Intrinsic Antibacterial Activity of Xeruborbactam *In Vitro*: Assessing Spectrum and Mode of Action

**DOI:** 10.1128/aac.00879-22

**Published:** 2022-09-14

**Authors:** Dongxu Sun, Ruslan Tsivkovski, Joe Pogliano, Hannah Tsunemoto, Kirk Nelson, Debora Rubio-Aparicio, Olga Lomovskaya

**Affiliations:** a Qpex Biopharma, Inc., San Diego, California, USA; b Linnaeus Biosciences, Inc., San Diego, California, USA; c University of California, San Diego, San Diego, California, USA

**Keywords:** *Acinetobacter*, *Enterobacterales*, PBP binding, xeruborbactam, beta-lactam antibiotics, enhancer

## Abstract

Xeruborbactam (formerly QPX7728) is a cyclic boronate inhibitor of numerous serine and metallo-beta-lactamases. At concentrations generally higher than those required for beta-lactamase inhibition, xeruborbactam has direct antibacterial activity against some Gram-negative bacteria, with MIC_50_/MIC_90_ values of 16/32 μg/mL and 16/64 μg/mL against carbapenem-resistant *Enterobacterales* and carbapenem-resistant Acinetobacter baumannii, respectively (the MIC_50_/MIC_90_ values against Pseudomonas aeruginosa are >64 μg/mL). In Klebsiella pneumoniae, inactivation of OmpK36 alone or in combination with OmpK35 resulted in 2- to 4-fold increases in the xeruborbactam MIC. In A. baumannii and P. aeruginosa, AdeIJK and MexAB-OprM, respectively, affected xeruborbactam’s antibacterial potency (the MICs were 4- to 16-fold higher in efflux-proficient strains). In Escherichia coli and K. pneumoniae, the 50% inhibitory concentrations (IC_50_s) of xeruborbactam’s binding to penicillin-binding proteins (PBPs) PBP1a/PBP1b, PBP2, and PBP3 were in the 40 to 70 μM range; in A. baumannii, xeruborbactam bound to PBP1a, PBP2, and PBP3 with IC_50_s of 1.4 μM, 23 μM, and 140 μM, respectively. Treating K. pneumoniae and P. aeruginosa with xeruborbactam at 1× and 2× MIC resulted in changes of cellular morphology similar to those observed with meropenem; the morphological changes observed after treatment of A. baumannii were consistent with inhibition of multiple PBPs but were unique to xeruborbactam compared to the results for control beta-lactams. No single-step xeruborbactam resistance mutants were obtained after selection at 4× MIC of xeruborbactam using wild-type strains of E. coli, K. pneumoniae, and A. baumannii; mutations selected at 2× MIC in K. pneumoniae did not affect antibiotic potentiation by xeruborbactam through beta-lactamase inhibition. Consistent with inhibition of PBPs, xeruborbactam enhanced the potencies of beta-lactam antibiotics even against strains that lacked beta-lactamase. In a large panel of KPC-producing clinical isolates, the MIC_90_ values of meropenem tested with xeruborbactam (8 μg/mL) were at least 4-fold lower than those in combination with vaborbactam at 64 μg/mL, the concentration of vaborbactam that is associated with complete inhibition of KPC. The additional enhancement of the potency of beta-lactam antibiotics beyond beta-lactamase inhibition may contribute to the potentiation of beta-lactam antibiotics by xeruborbactam.

## INTRODUCTION

In general, clinically used beta-lactamase inhibitors lack antibacterial activity of their own, with some exceptions: both clavulanic acid and sulbactam (but not tazobactam) have moderate activity against Neisseria spp., Haemophilus influenzae, Bacteroides, and other anaerobes. Sulbactam has relatively potent intrinsic activity against Acinetobacter spp. (1, 2). Avibactam (but not relebactam or vaborbactam) has activity against select strains of *Enterobacterales* (3). In all cases studied, this direct antibacterial activity of various BLIs was attributed to inhibition of penicillin-binding proteins (PBPs), which is not surprising given the structural relationship between PBPs and beta-lactamases (4). Specifically, sulbactam was shown to inhibit PBP1 and PBP3 in Acinetobacter baumannii (5) and avibactam had the highest affinity to PBP2 from Escherichia coli, H. influenzae, and Pseudomonas aeruginosa (6). Several other diazabicyclooctane (DBO) beta-lactamase inhibitors (BLIs) that are in various stages of clinical development, including zidebactam, durlobactam, ETX1317, and nacubactam, are also specific inhibitors of PBP2 but have more potency than avibactam (7–10). When these BLIs are combined with beta-lactams that inhibit other PBPs, such as PBP1 and/or PBP3, they demonstrate a beta-lactam enhancer effect, in addition to beta-lactamase inhibition, that is based on simultaneous inhibition of multiple PBPs (11).

**FIG 1:**
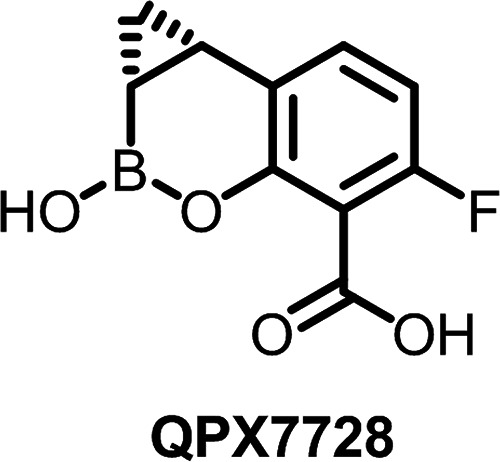
Xeruborbactam (QPX7728).

Xeruborbactam (formerly known as QPX7728) (Fig. 1) is a cyclic boronate inhibitor of numerous serine and metallo-beta-lactamases (12, 13). Examples of serine beta-lactamases inhibited by xeruborbactam with nanomolar potency include carbapenemases, such as class A KPC and class D OXA enzymes from *Enterobacterales* (e.g., OXA-48) and A. baumannii (e.g., OXA-23/OXA-40), and various class A and class C beta-lactamases that hydrolyze cephalosporins, penicillins, and monobactams (14, 15). Xeruborbactam was shown to enhance the activity of multiple beta-lactam antibiotics against various Gram-negative bacteria, including carbapenemase or extended-spectrum beta-lactamase (ESBL)-producing *Enterobacterales* (16, 17), carbapenem-resistant Acinetobacter baumannii (18), and multidrug-resistant Pseudomonas aeruginosa (19). Unlike other notable cyclic boronates, such as vaborbactam or the dual serine/metallo-beta-lactamase inhibitor taniborbactam (20), xeruborbactam possesses modest direct antibacterial activity. The objective of this study was to investigate the mechanism of the antibacterial activity of xeruborbactam.

## RESULTS AND DISCUSSION

### Xeruborbactam has low-potency intrinsic antibacterial activity.

**FIG 2:**
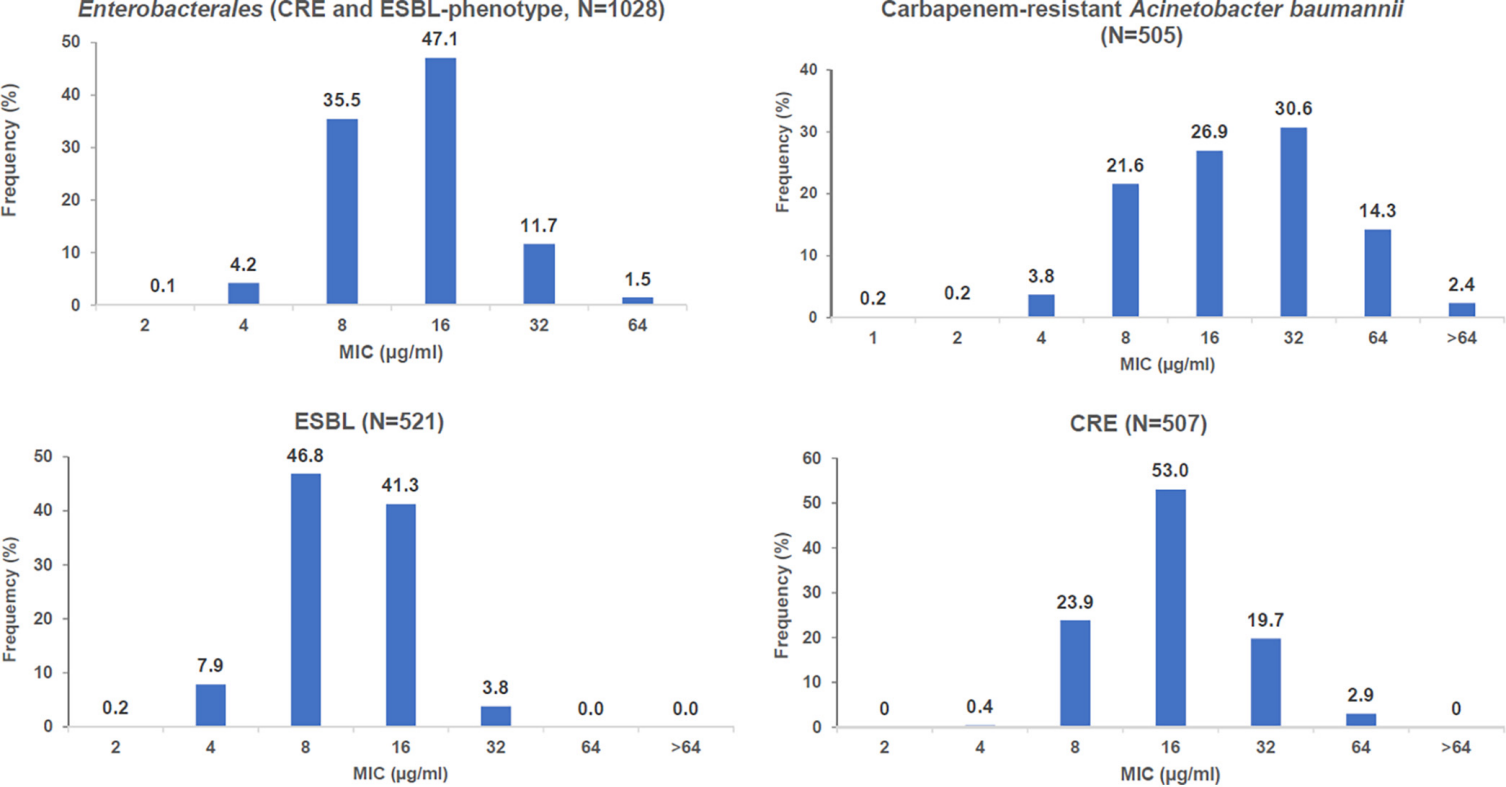
Distribution of xeruborbactam MICs in *Enterobacterales* (ESBL phenotype and or/carbapenem-resistant isolates) and carbapenem-resistant Acinetobacter baumannii. ESBL phenotype was defined as ceftazidime or aztreonam MIC values of ≥1 μg/mL; carbapenem resistance was identified based on molecular analysis (presence of the proven known carbapenemases) or meropenem MIC values of ≥4 μg/mL.

Besides its beta-lactamase inhibitory action, xeruborbactam has low-potency direct antibacterial activity. Xeruborbactam MICs against 1,028 isolates of *Enterobacterales* (including 507 carbapenem-resistant *Enterobacteriaceae* [CRE] isolates and 521 isolates with an ESBL phenotype) ranged from 2 μg/mL to 64 μg/mL, with MIC_50_/MIC_90_ values of 16/32 μg/mL and a modal MIC of 16 μg/mL (Table 1, Fig. 2). Per-species analysis indicated slight variations in xeruborbactam potency; for example, based on the MIC_90_ and the highest xeruborbactam MIC observed, Klebsiella pneumoniae and Enterobacter cloacae species complex isolates (MIC_90_ of 32 μg/mL and a MIC of 64 μg/mL being the highest) appeared to be 2-fold more resistant than Escherichia coli and Klebsiella oxytoca isolates (MIC_90_ of 16 μg/mL and a MIC of 32 μg/mL being the highest). Among K. pneumoniae isolates, xeruborbactam appeared to be 2-fold more potent against the subgroup of strains with an ESBL phenotype than against CRE strains. Against 505 isolates of carbapenem-resistant A. baumannii, xeruborbactam MICs were in the range of 1 to >64 μg/mL, with MIC_50_/MIC_90_ values of 16/64 μg/mL and a modal MIC of 32 μg/mL (Table 1, Fig. 2). Xeruborbactam was less potent against P. aeruginosa; 94% of the 506 clinical isolates tested had MICs of ≥64 μg/mL (MIC_50_/MIC_90_ values of >64/>64 μg/mL). The MICs of the remaining 6% of strains ranged from 8 μg/mL to 32 μg/mL. Xeruborbactam was also tested in a small panel of Gram-positive bacteria that included Enterococcus faecalis (*n* = 16), Streptococcus pneumoniae (*n* = 18), and Staphylococcus aureus (methicillin-susceptible S. aureus [MSSA; *n* = 12] and methicillin-resistant S. aureus [MRSA; *n* = 13]) isolates. All MSSA strains were inhibited with xeruborbactam at 4 to 8 μg/mL (MIC_50_/MIC_90_ values of 8/8 μg/mL), but the MICs increased to >32 μg/mL for all the MRSA strains. The xeruborbactam MICs against all the E. faecalis strains were >32 μg/mL, and they ranged from 4 μg/mL to >32 μg/mL (MIC_50_/MIC_90_ of 32/>32 μg/mL) against S. pneumoniae strains (Table S3 in the supplemental material).

**TABLE 1:** *In vitro* antibacterial activity of xeruborbactam against *Enterobacterales*, carbapenem-resistant Acinetobacter baumannii, and Pseudomonas aeruginosa

Organism(s) or resistance mechanism	No. of isolates	Value (μg/mL) for:		Range
		MIC_50_	MIC_90_	
*Enterobacterales*	1,028	16	32	2 to 64
Klebsiella pneumoniae	575	16	32	4 to 64
Klebsiella pneumonia CRE^*a*^	418	16	32	4 to 64
KPC	163	16	32	8 to 64
OXA-48-like	89	16	64	8 to 64
Non-carbapenemase producing	40	16	32	8 to 64
Metallo-beta-lactamase	126	16	32	8 to 32
Klebsiella pneumonia ESBL phenotype^*b*^	157	16	16	4 to 32
Escherichia coli	334	8	16	2 to 32
Enterobacter cloacae species complex	45	16	32	4 to 64
Klebsiella oxytoca	23	8	16	8 to 32
Proteus mirabilis	23	16	32	8 to 16
Serratia marcescens	10	32	32	16 to 64
Others^*c*^	18	16	32	8 to 64
Carbapenem-resistant Acinetobacter baumannii	505	16	64	1 to >64
Pseudomonas aeruginosa	506	>64	>64	8 to >64

a CRE, carbapenem-resistant *Enterobacteriaceae.*

b ESBL phenotype was defined as a MIC of ≥1 μg/mL of either ceftazidime or aztreonam.

c Other *Enterobacterales* included Citrobacter amalonaticus/farmeri (*n* = 1), Citrobacter freundii species complex (*n* = 7), Citrobacter koseri (*n* = 3), Enterobacter hormaechei (*n* = 2), Klebsiella aerogenes (*n* = 4), and Providencia stuartii (*n* = 1).

### Antibacterial potency of xeruborbactam is affected by intrinsic resistance mechanisms in *Enterobacterales*, Pseudomonas aeruginosa, and Acinetobacter baumannii.

Isogenic laboratory mutants were used to investigate the effects of efflux and porin mutations on the direct antibacterial activity of xeruborbactam and DBO BLIs avibactam, zidebactam, and durlobactam. Against wild-type strains of Escherichia coli or Klebsiella pneumoniae, the antibacterial potencies of xeruborbactam (8 to 16 μg/mL) were similar to those of avibactam and nacubactam and lower than those of zidebactam and durlobactam (0.125 to 0.5 μg/mL) (Table 2). Inactivation or overexpression of the major efflux pump AcrAB-TolC did not affect the potency of xeruborbactam in either of these organisms. Among the BLIs compared, zidebactam was the only one mildly (4-fold) affected by the overexpression of AcrAB in E. coli. In K. pneumoniae isolates, deletion of *ompK35* had no effect on either xeruborbactam or DBO BLI MICs (compare strain KPM1026a to strain KPM2600). Inactivation of *ompK36* alone or in combination with *ompK35* resulted in a 2-fold increase in the xeruborbactam MIC (compare strain KPM1026a to strains KPM2040 and KPM2613). These same mutations resulted in higher increases in DBO BLI MICs (>64 μg/mL for avibactam, nacubactam, and zidebactam). The highest, or 4-fold, MIC increase for xeruborbactam was observed for strain KPM1171 (MIC of 64 μg/mL), which both lacked porins and overexpressed AcrAB. This strain also had the highest MICs for DBO BLIs (Table 2).

**TABLE 2:** *In vitro* activity of various BLIs against isogenic strains of Gram-negative bacteria

Strain	Genotype (phenotype)	MIC (mg/L) of:							
		Xeruborbactam	Avibactam	Nacubactam	Zidebactam	Durlobactam	Meropenem	Cefepime	Aztreonam
Escherichia coli strains									
ECM5497	Wild type	8	16	4	0.125	0.5	0.03	0.03	0.03
ECM6001	*ΔacrB*	8	16	4	0.125	0.5	0.03	0.03	0.03
ECM6502	*marR* (AcrAB overexpression)	8	8	2	0.5	0.25	0.06	0.125	0.25
Klebsiella pneumoniae strains									
KPM1026a	Wild type	16	16	8	0.25	0.5	0.03	0.03	0.03
KPM1027	*ramR*	16	16	4	0.25	0.5	0.06	0.25	0.25
KPM2600	Δ*ompK35*	16	16	8	0.25	0.5	0.06	0.25	0.125
KPM2040	*ompK36_2040*	16	256	>64	>64	4	0.125	0.125	0.06
KPM2613	Δ*ompK35 ompK36_2040*	32	256	>64	>64	8	0.25	0.25	0.25
KPM1171	*ramR ompK36_1171*	64	>256	>64	>64	32	2	2	0.5
KPM2696	Δ*acrB*	16	16	2	0.25	0.25	0.03	0.03	0.03
Pseudomonas aeruginosa strains									
PAM1020	Wild type	256	>256	128	2	64	0.25	0.5	4
PAM1032	*nalB* (MexAB-OprM overexpression)	>256	>256	128	4	>256	1	4	16
PAM1033	*nfxB* (MexCD-OprJ overexpression)	128	32	32	0.125	4	0.25	4	1
PAM1034	*nfxC* (MexEF-OprN overexpression)	256	>256	128	2	128	0.5	0.5	4
PAM1106	*mexA*::Tet	16	128	128	2	8	0.06	0.25	0.125
PAM1154	*oprM*::Hg	16	128	128	2	8	0.06	0.125	0.125
Acinetobacter baumannii strains									
AB1007	Wild type	16	>256	>256	>256	64	0.25	4	32
ACM1027	*adeN* (AdeIJK overexpression)	32	>256	>256	>256	128	1	8	64
ACM1030	*adeS* (AdeABC overexpression)	16	>256	>256	>256	64	0.5	32	16
ACM1013	Δ*adeIJK*	4	>256	>256	>256	32	0.125	4	1
ACM1014	Δ*adeABC*	16	>256	>256	>256	64	0.125	1	16
ACM1015	Δ*adeIJK* Δ*adeABC*	4	>256	>256	>256	16	0.06	0.25	1

The analysis of 453 clinical isolates of K. pneumoniae enriched in strains with porin mutations (17) was consistent with the results obtained with laboratory mutants. The functional status of OmpK36 appeared to be the major factor affecting xeruborbactam MIC values (Table S4). Out of 65 isolates with a nonfunctional OmpK36 (insertion, frameshift, and nonsense mutations in the *ompK36* gene or its decreased expression), 60 isolates (92%) had xeruborbactam MIC values of ≥32 μg/mL; out of 160 isolates with a two-amino-acid insertion (Gly134Asp135, GD repeat) in the L3 loop of OmpK36 (resulting in a partially functional OmpK36 with a constricted channel), 120 isolates (75%) had xeruborbactam MIC values of ≥32 μg/mL; and out of 228 isolates with the full-length OmpK36, only 12 isolates (5.3%) had xeruborbactam MIC values of ≥32 μg/mL, and the MIC values of ~95% of these isolates were in an 8- to 16-μg/mL range. As was shown using the isogenic panel, the functional status of OmpK35 alone did not appear to significantly affect xeruborbactam MICs; 31.4% and 49% of isolates with full-length or nonfunctional OmpK35, respectively, had xeruborbactam MIC values of ≥32 μg/mL. While OmpK35 inactivation did not increase the proportion of strains with increased xeruborbactam MIC values in the subset of strains with a full-length OmpK36, it appeared to do so in the subset of strains with a nonfunctional OmpK36 (Table S4). The limited analysis of E. coli and E. cloacae isolates performed to date was also consistent with the major role of OmpK36/OmpC: all the strains with the highest xeruborbactam MIC values had both nonfunctional OmpK36/OmpC and nonfunctional OmpK35/OmpF (Table 3). We used the tigecycline MIC as a phenotypic test for the presence of regulatory mutations that simultaneously decreased the expression of *ompK35* and increased the expression of the *acrAB* efflux operon (21, 22); increased efflux appeared to play some role but a rather minimal one (compare E. cloacae strain ECL1057 to strain ECL1079 and K. pneumoniae strains KP1074 and KP1093 to strain KP1598). Based on these results, we hypothesize that the distribution of xeruborbactam MICs in *Enterobacterales* isolates correlates with the distribution of specific *ompK36*/*ompC* and double *ompK36*/*ompC* and *ompK35*/*ompF* mutations alone or in combination with regulatory efflux mutations.

**TABLE 3:** Effects of intrinsic resistance mechanisms on xeruborbactam MICs in clinical isolates of Klebsiella pneumoniae, Escherichia coli, and Enterobacter cloacae

Strain	Beta-lactamases	OmpK35^*a*^		OmpK36^*a*^		MIC (μg/mL) of:	
		Description	Functional status	Description	Functional status	Xeruborbactam	Tigecycline^*b*^
Escherichia coli strains							
EC1081	CTX-M-15, OXA-1	Full length	FN	Full length	FN	8	0.5
EC1085	None	Full length	FN	Full length	FN	8	0.25
EC1121	CMY-6, CTX-M-15, NDM-1, OXA-2, TEM-1B-like	FS from aa 31	NF	Full length	FN	8	0.25
EC1088	KPC-3, OXA-9, TEM-1A	Full length	FN	Full length	FN	8	0.125
EC1097	KPC-3, TEM-1B	Full length	FN	Full length	FN	8	1
EC1098	CMY-6, NDM-1, OXA-2, TEM-1A-like	FS from aa 31	NF	Full length	FN	8	1
EC1078	CMY-2, CTX-M-14, TEM-1B	FS from aa 251	NF	E130stop	NF	32	0.5
EC1119	CTX-M-15 OXA-1	FS from aa 213	NF	IS1414 at nt 756	NF	32	4
Klebsiella pneumoniae strains							
KP1070	KPC-2, SHV-11, SHV-12, TEM-1	FS from aa 42	NF	Full length	FN	8	1
KP1274	SHV-30, VIM-1	Full length	FN	Full length	FN	8	1
KP1004	KPC-2, TEM-1, SHV-11	FS from aa 42	NF	Full length	FN	16	1
KP1251	KPC-3 TEM SHV	FS from aa 42	NF	Full length, R191L	FN	16	1
KP1074	KPC-3, SHV-11, TEM-1	FS from aa 42	NF	GD	PFN	32	0.5
KP1093	KPC-3 TEM-1 SHV-11	FS from aa 42	NF	GD	PFN	32	1
KP1463	KPC-2, CTX-M-65, TEM-1, SHV-11	FS from aa 29	NF	TGA at aa 94	NF	64	1
KP1598	KPC-2, SHV-11, CTX-M-14	FS from aa 29	NF	GD	PFN	64	4
KP1469	KPC-33, CTX-M-15, OXA-1, TEM-1, SHV-28	Full length, low expression	NF	FS from aa 5	NF	64	4
Enterobacter cloacae strains							
ECL1126	KPC-2, TEM	Full length	FN	Full length	FN	16	2
ECL1131	KPC-4, TEM, OXA-1	FS from aa 19	NF	Full length	FN	16	2
ECL1057	NDM-1, TEM-1, CTX-M-15	Full length	FN	FS at aa 210	NF	64	8
ECL1079	KPC-3, TEM	Q60stop	NF	TAA at codon 78	NF	64	4

a aa, amino acid; FS, frameshift; GD, insertion of two amino acids, Gly134Asp135, in the L3 loop of OmpK36, resulting in a partially functional OmpK36 with a constricted channel; FN, functional; NF, nonfunctional; nt, nucleotide; PFN, partially functional.

b Increased tigecycline MIC values are associated with regulatory mutations that simultaneously decrease the expression of *ompK35* and increase efflux by the AcrAB-TolC efflux pump (21, 22).

In Pseudomonas aeruginosa, inactivation of the major constitutively expressed efflux pump MexAB-OprM resulted in a 16-fold reduction of the xeruborbactam MIC (from 256 μg/mL to 16 μg/mL) (Table 2). This is in contrast to a minimal effect of MexAB-OprM on the BLI activity of xeruborbactam (23). It is speculated that at the relatively low periplasmic concentration of xeruborbactam that is required for beta-lactamase inhibition, the need for efflux is negligible, but efflux becomes more significant at the higher concentrations required for antibacterial effects. Similar effects of MexAB-OprM inactivation on MIC reduction were seen for durlobactam (from 64 μg/mL to 8 μg/mL) and, potentially, avibactam (from >256 μg/mL to 128 μg/mL). Zidebactam and nacubactam did not appear to be affected by MexAB-OprM. Overexpression of other clinically relevant efflux pumps, MexCD-OprJ and MexEF-OprN, did not increase xeruborbactam MICs; the MIC was decreased 2-fold against *mexCD-oprJ*-overexpressing strain PAM1033. Interestingly, the MIC reductions (4-fold to >32-fold) against this strain were greater for DBO BLIs. Of note, earlier studies have demonstrated that strains that overexpress MexCD-OprJ become hypersusceptible to many beta-lactam antibiotics (24, 25). Based on the results with the isogenic efflux panel, it is likely that strains of P. aeruginosa with xeruborbactam MIC values at or below 32 μg/mL (30 strains in this analysis) have decreased expression or inactivation of the MexAB-OprM efflux pump. In fact, 29 of 30 such strains (96.7%) had aztreonam MIC values alone or with xeruborbactam (fixed concentration of 4 μg/mL or 8 μg/mL) in the range of 0.125 to 2 μg/mL, which is consistent with the decreased activity of the MexAB-OprM efflux system (Table 2, Table S6) (using the aztreonam-xeruborbactam MIC in addition to the MIC of aztreonam alone allowed the assessment of efflux activity even in the presence of a beta-lactamase). Ninety-six percent of isolates with xeruborbactam MIC values of ≥64 μg/mL had aztreonam MIC values alone or with xeruborbactam of ≥4 μg/mL, which is associated with either wild-type or increased activity of the MexAB-OprM efflux pump.

In Acinetobacter baumannii, AdeIJK-mediated efflux appeared to affect the intrinsic antibacterial activity of xeruborbactam (2-fold MIC increase or 4-fold MIC decrease upon *adeIJK* overexpression or inactivation, respectively). Similar effects were also observed for durlobactam. The MICs of other DBOs against A. baumannii were >256 μg/mL irrespective of the status of efflux operons (Table 2).

**FIG 3:**
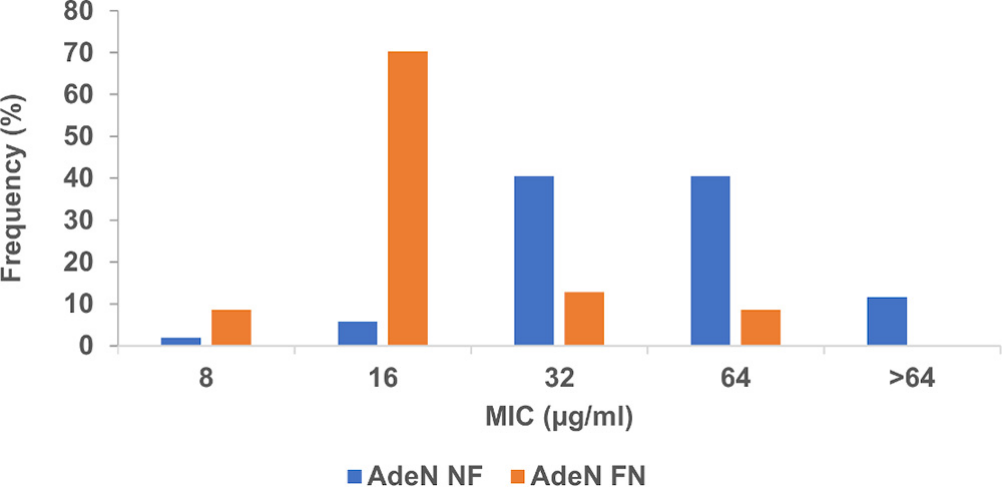
Distribution of xeruborbactam MICs in clinical isolates of carbapenem-resistant A. baumannii based on the functional status of AdeN, the negative regulator of the *adeIJK* efflux operon. The analysis is based on 52 isolates containing nonfunctional AdeN due to insertion, deletion, frameshift, and nonsense mutations in the *adeN* gene (AdeN NF) and 47 isolates containing the wild-type, functional copy of *adeN* (AdeN FN).

To assess the relevance of the AdeIJK-mediated efflux in clinical isolates of carbapenem-resistant A. baumannii, we compared the distribution of xeruborbactam MICs in 52 isolates with inactivated *adeN* (due to insertion, deletion, frameshift, and nonsense mutations) and in 47 isolates with the wild-type *adeN* gene (17). There was a significant degree of separation of MIC distributions, with higher proportions of strains with xeruborbactam MIC values of ≥32 μg/mL and ≤16 μg/mL among the strains with the nonfunctional and functional AdeN, respectively (Fig. 3). These data are consistent with the increased xeruborbactam MIC values due to AdeIJK-mediated efflux in clinical isolates of carbapenem-resistant A. baumannii.

### Xeruborbactam has time-dependent killing of Klebsiella pneumoniae.

**FIG 4:**
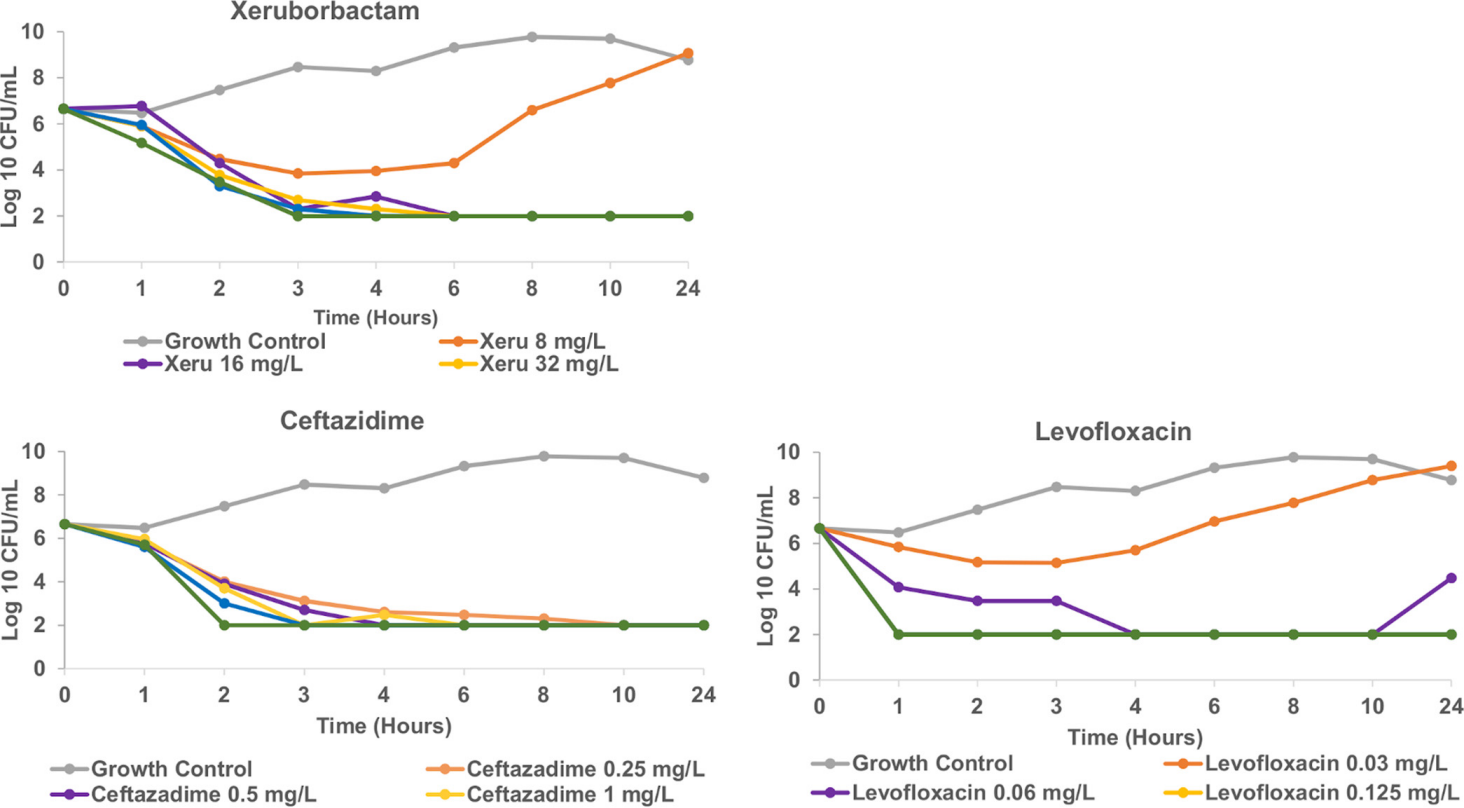
Kinetics of killing of Klebsiella pneumoniae strain KPM1026a by xeruborbactam, ceftazidime, and levofloxacin. The MICs for xeruborbactam, ceftazidime, and levofloxacin were 16 μg/mL, 0.25 μg/mL and 0.06 μg/mL, respectively. The limit of detection was 10^2^ CFU/mL.

The xeruborbactam minimal bactericidal concentration (MBC) was the same or not more than 2-fold higher than its MIC against strain ECM5497 of E. coli (the MIC and MBC were both 8 μg/mL), strain KPM1026a of K. pneumoniae (the MIC and MBC were both 16 μg/mL), strain PAM1154 of P. aeruginosa (MIC and MBC values of 16 μg/mL and 32 μg/mL, respectively), and strain AB1007 of A. baumannii (the MIC and MBC were both 16 μg/mL), indicating bactericidal activity. Time-kill studies performed with strain KPM1026a demonstrated time-dependent, concentration-independent bacterial killing similar to that observed with ceftazidime (Fig. 4).

### Xeruborbactam binds to penicillin-binding proteins.

**FIG 5:**
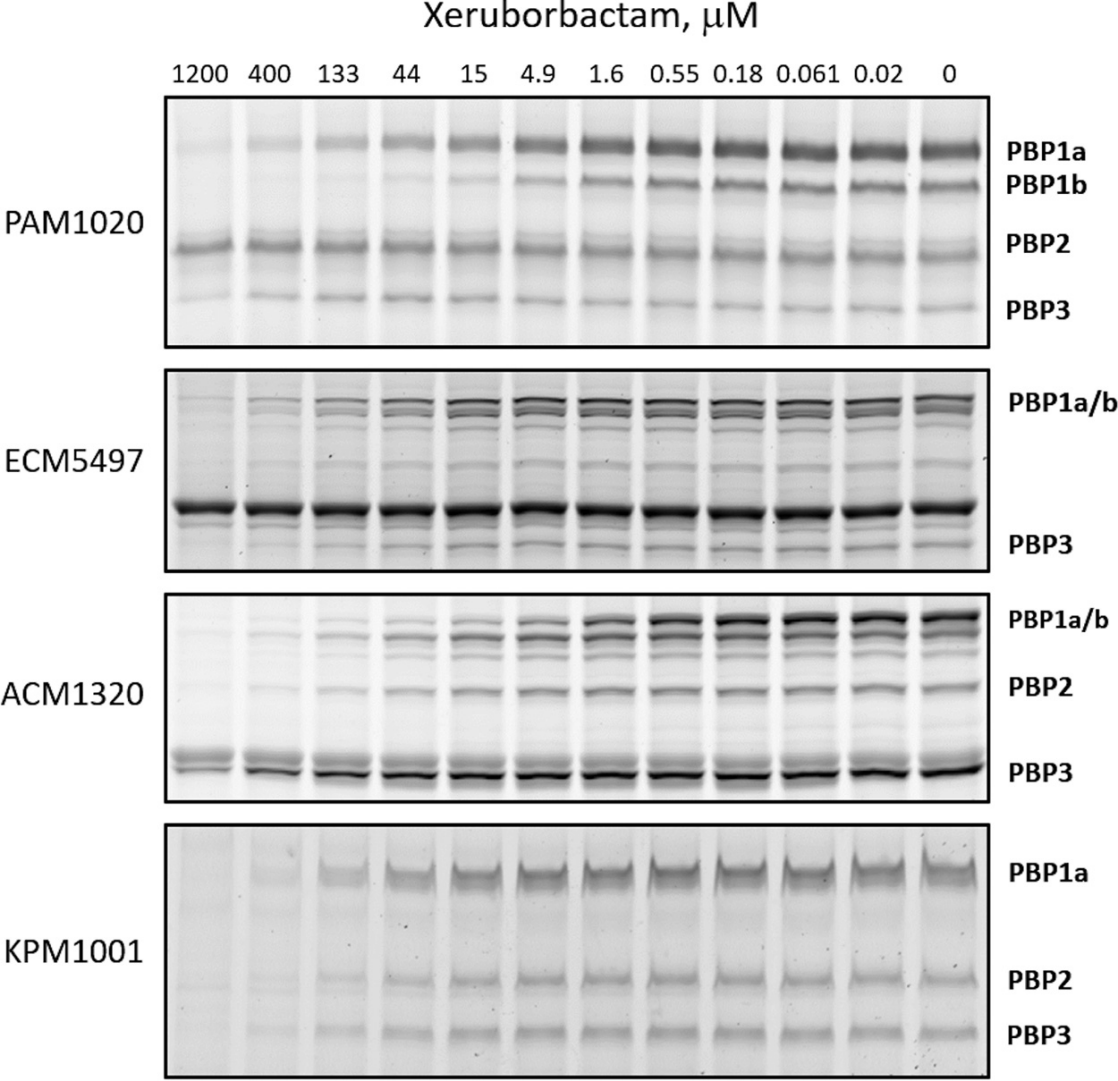
Inhibition of PBP activity by xeruborbactam in bacterial membrane preparations. Incubation of bacterial membrane preparations with control beta-lactams was performed for 10 min at 30°C; xeruborbactam was incubated with membrane preparations for 1 h at 30°C. Bocillin FL fluorescent probe was added, and the reaction mixture was incubated for an additional 10 min at 30°C in the case of beta-lactams or for 20 min on ice (0°C) in the case of xeruborbactam, to prevent displacement of xeruborbactam from its complex by the Bocillin probe.

The relative binding affinities of xeruborbactam with the major PBPs in total membrane preparations from E. coli, K. pneumoniae, A. baumannii, and P. aeruginosa were assessed using the gel-based Bocillin FL competition assay. No detectable interactions of xeruborbactam with PBPs were observed at concentrations up to 300 μM using experimental conditions under which the control beta-lactams meropenem, aztreonam, and amdinocillin showed measurable binding (Fig. S1); however, when the temperature for the fluorescent probe incubation step was reduced from 30°C to 0°C, xeruborbactam binding with PBPs was detected (Fig. 5). Of note, the same modification of the PBP binding assay was previously used to detect the binding of the monobactam tigemonam to PBP3, in order to prevent the displacement of the PBP-bound tigemonam by the radiolabeled penicillin (26). Xeruborbactam interacted with several detectable PBPs from E. coli and K. pneumoniae (PBP1a/PBP1b, PBP2, and PBP3) with modest but similar relative affinities, with IC_50_ values in the 40 to 70 μM range (Table 4). In the case of A. baumannii, the highest affinity of xeruborbactam was with PBP1a (IC_50_ = 1.4 ± 0.5 μM), followed by PBP2 (IC_50_ = 23 ± 2 μM) and PBP3 (IC_50_ = 135 ± 40 μM). For P. aeruginosa PBPs, the highest affinity was with PBP1a (IC_50_ = 1.9 ± 0.2 μM), followed by PBP1b (IC_50_ = 7.1 ± 0.8 μM) and PBP2 (IC_50_ = 370 ± 120 μM). Meropenem had a different pattern of PBP inhibition, with the highest value for PBP2, in agreement with previous reports (27–29), followed by PBP3 in E. coli, P. aeruginosa, and K. pneumoniae and PBP1a in A. baumannii (Table 4).

**TABLE 4:** Relative binding affinities of xeruborbactam and meropenem with the major penicillin-binding proteins from Gram-negative bacteria

Strain	Compound	MIC (μg/mL) (μM)	Relative binding [mean IC_50_ ± SD or IC_50_ (μM)] of^*a*^:			
			PBP1a	PBP1b	PBP2	PBP3
Escherichia coli ECM5497 (MG1655)	Xeruborbactam	16 (72)	**47 ± 13**	**56 ± 18**	ND^*b*^	**73 ± 10**
	Meropenem	0.03 (0.08)	5.4 (4.4)^*d*^	21.1 (3.4)^*d*^	ND (**0.003**)^*b*^^,^^*d*^	0.46 (1.57)^*d*^
Klebsiella pneumoniae KPM1001 (ATCC 49816)	Xeruborbactam	16 (72)	**41 ± 6**	ND^*c*^	**51 ± 15**	**55 ± 8**
	Meropenem	0.03 (0.08)	6.6 (5.2)^*e*^	ND^*c*^	**0.003** (<0.02)^*e*^	**0.24** (0.31)^*e*^
Acinetobacter baumannii ACM1320 (ATCC 17978)	Xeruborbactam	16 (72)	**1.4 ± 0.5**	ND^*c*^	**23 ± 2**	140 ± 40
	Meropenem	0.25 (0.66)	**0.07** (<0.08)^*f*^	ND (**0.5**)^*c*^^,^^*f*^	**0.011** (<0.08)^*f*^	**0.19** (0.33)^*f*^
Pseudomonas aeruginosa PAM3034 (PAO1)	Xeruborbactam	16 (72)	**7.1 ± 0.8**	**1.9 ± 0.2**	370 ± 120	510 ± 120
	Meropenem	0.06 (0.16)	**0.17** (0.5)^*d*^	**0.23** (0.5)^*d*^	ND (**0.05**)^*d*^	**0.096** (0.08)^*d*^

a Various concentrations of xeruborbactam or meropenem were incubated with membrane proteins from E. coli, K. pneumoniae and A. baumannii in a gel-based, Bocillin FL competition assay. Bold font indicates potential inhibition of the indicated PBP that might occur at the MIC. ND, not determined.

b Not determined due to the presence of an overlapping nonspecifically labeled band on the gel.

c Not determined due to a missing band on the gel.

d Data are for E. coli MC1400 and for P. aeruginosa PAO1 from reference 28.

e Data are for K. pneumoniae ATCC 43816 from reference 47.

f Data are for A. baumannii ATCC 19606 from reference 27.

The observations of xeruborbactam’s interaction with various PBPs (albeit with modest affinity) suggest that its antibacterial activity is explained at least in part by PBP inhibition. In fact, the xeruborbactam MIC values for E. coli and K. pneumoniae were in the same range, 72 μM (16 μg/mL), as the IC_50_ values for inhibition of multiple PBPs. While the xeruborbactam MIC for the strain of A. baumannii used in PBP-binding experiments was 72 μM (16 μg/mL), it was 18 μM (4 μg/mL) for the pumpless strain, in the range of A. baumannii PBP1a and PBP2 inhibition.

### Xeruborbactam-induced changes in cellular morphology are consistent with inhibition of multiple PBPs.

**FIG 6:**
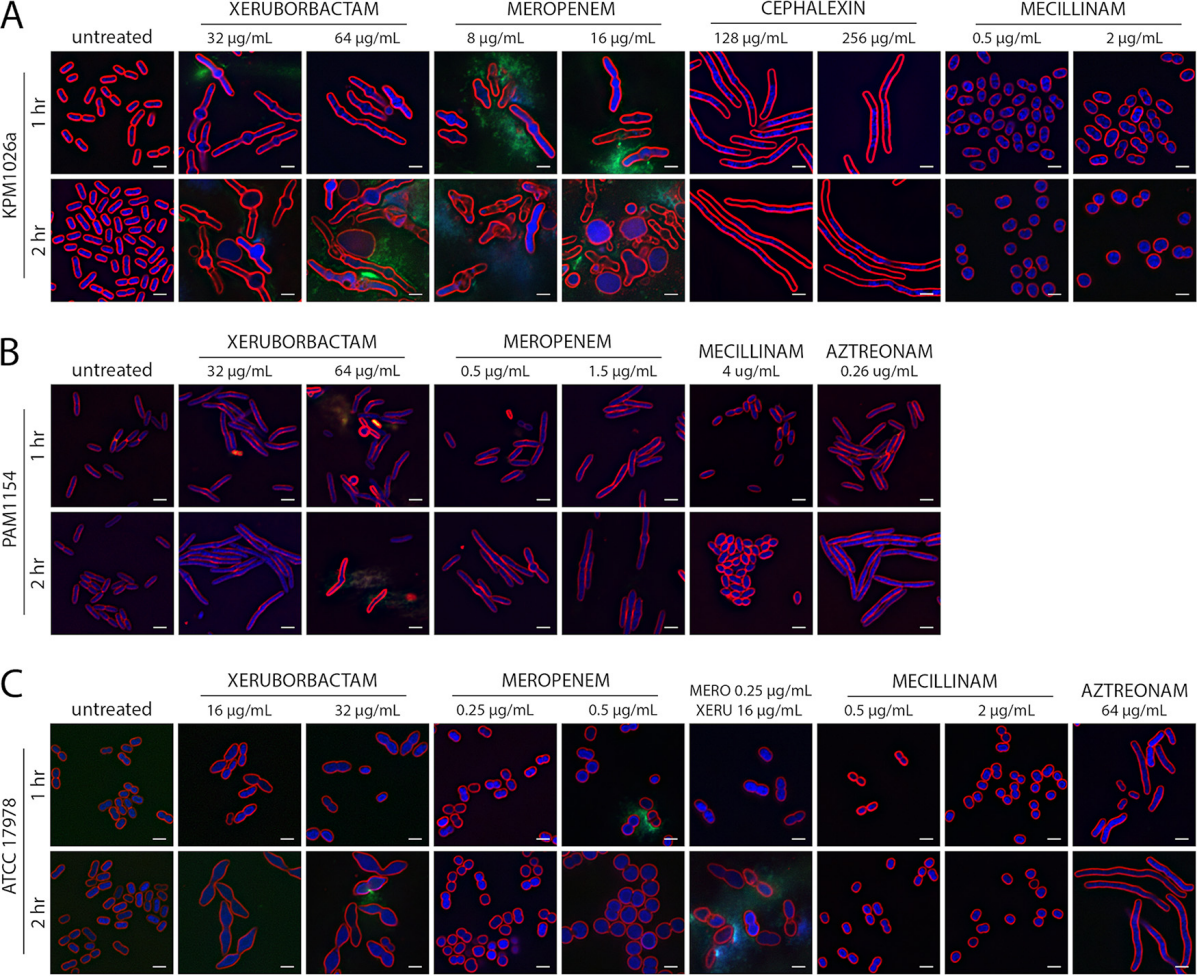
Morphologies of cells of K. pneumoniae, A. baumannii, and P. aeruginosa after treatment with xeruborbactam and control antibiotics. Klebsiella pneumoniae strain KPM1026a (A), Pseudomonas aeruginosa strain PAM1154 (B), and Acinetobacter baumannii strain ATCC 17978 (C) were treated with 1× and 2× MIC xeruborbactam for 1 and 2 h at 30°C. Meropenem, cephalexin, mecillinam (the USAN name amdinocillin is used in the text), and aztreonam were used as antibiotic controls. Cells were stained with FM4-64 (red, membrane stain), DAPI (blue, membrane-permeable DNA stain), and SYTOX green (green, membrane-impermeable DNA stain). Scale bar = 2 μm. The MICs for the tested strains were as follows. Strain KPM1026a measured at 30°C: xeruborbactam, 32 μg/mL; meropenem, 0.06 μg/mL; cephalexin, 128 μg/mL; and amdinocillin, 128 μg/mL. Strain PAM1154: xeruborbactam, 32 μg/mL; meropenem, 0.25 μg/mL; aztreonam, 0.25 μg/mL; and amdinocillin, 128 μg/mL. Strain ATCC 17978: xeruborbactam, 16 μg/mL; meropenem, 0.25 μg/mL; aztreonam, 32 μg/mL; and amdinocillin, 128 μg/mL.

PBPs function as components of multiprotein complexes that synthesize and remodel peptidoglycan (30, 31). Consequently, inhibition of PBPs induces specific morphological changes in cells based on particular roles they play in coordinating division and elongation (29, 32). It was previously demonstrated that traditional PBP inhibitors, beta-lactam antibiotics, with the same PBP-binding preferences cause similar morphological variations, such as ovoid morphology due to inhibition of PBP2 (part of the elongasome) (amdinocillin), filamentous morphology due to inhibition of PBP3 (part of the divisome) (cephalexin and aztreonam) (33), or filaments that are swollen at midcell due to simultaneous inhibition of PBP2/PBP3 (meropenem) (28). To gain insight into the relevance of xeruborbactam’s PBP binding for its antibacterial mode of action, we investigated the effects of xeruborbactam and several control beta-lactams on cellular morphology using the previously described methodology (34). In these experiments, cells of K. pneumoniae strain KPM1026a, A. baumannii strain ATCC 17978, and P. aeruginosa strain PAM1154 (lacks major efflux pumps) were treated with xeruborbactam at 1× and 2× MIC. K. pneumoniae KPM1026a cells treated with xeruborbactam for 1 h had both increased cell length relative to that of the untreated controls and bulges that developed at midcell. By 2 h, the swelling at the midcell was more pronounced, and several spheroplasts could be observed, as well as cell lysis. This phenotype mimicked the effect of meropenem and was consistent with inhibition of multiple PBPs (32). As noted above and shown by the results in Table 4, both meropenem and xeruborbactam at MIC are expected to inhibit PBP2 and PBP3, which might lead to cell elongation and midcell swelling. In comparison, treatment of strain KPM1026a with cephalexin or amdinocillin led only to filamentation or cell rounding, indicating PBP3 or PBP2 inhibition, respectively (Fig. 6A).

Treatment of P. aeruginosa strain PAM1154 (in which *oprM* is inactivated) with xeruborbactam at 1× or 2× MIC (32 or 64 μg/mL, respectively) caused morphological changes similar to those resulting from meropenem treatment, leading to filamentation and bulge formation at midcell and eventual cell lysis from the midpoint, as observed previously with K. pneumoniae (Fig. 6B).

Treatment of cells of A. baumannii strain ATCC 17978 with xeruborbactam caused cell shape deformation, from coccobacilli to a football shape, relative to the cell shape of its untreated control (Fig. 6C). In comparison, meropenem treatment at 0.25 μg/mL and 0.5 μg/mL (1× and 2× MIC, respectively) caused swelling and rounding but not elongation. Aztreonam treatment at 2× MIC led to an increase in cell length, and amdinocillin at 2 μg/mL (below the MIC of >256 mg/L) caused bacterial cell rounding but not swelling.

We also tested treatment of A. baumannii ATCC 17978 with 1× MIC of both xeruborbactam (16 μg/mL) and meropenem (0.25 μg/mL) in combination. After 2 h of treatment with xeruborbactam and meropenem, the bacteria had a mixed phenotype with characteristics of both antibiotic treatments. The cells were rounder than for the xeruborbactam treatment alone, more reminiscent of meropenem treatment, but still had slightly pointed cell poles, which could be observed during xeruborbactam treatment alone (Fig. 6C). These results show that xeruborbactam treatment has a distinct morphological effect in A. baumannii strain ATCC 17978 compared to the effects of the control antibiotics meropenem, amdinocillin, and aztreonam. It is conceivable that this unique phenotype may be due to various affinities of xeruborbactam for different PBPs.

Overall, the results of cytological profiling indicated that inhibition of multiple PBPs was at least one of the mechanisms for the antibacterial activity of xeruborbactam against *Enterobacterales*, Pseudomonas aeruginosa, and Acinetobacter baumannii.

### Mutations conferring low-level resistance to the antibacterial activity of xeruborbactam are consistent with its targeting of cell wall biogenesis.

Recently, PBP3 substitutions consisting of a four-amino-acid insertion of YRIN or YRIK after position P333 were associated with increased resistance to PBP3 inhibitors alone or in combination with avibactam in clinical isolates of E. coli (35, 36). Several such isolates from our collection were tested for susceptibility to xeruborbactam and control compounds. While these PBP3 mutants did have increased aztreonam-avibactam MIC values (aztreonam targets PBP3 with high selectivity), no increase in xeruborbactam MIC values was observed (Table S5). In our collection of clinical isolates with available whole-genome sequences, we also identified several strains with substitutions in PBP2 (T331P [a change of T to P at position 331], M574I, and M543T) that had increased MIC values of the highly selective PBP2 inhibitors zidebactam and/or durlobactam. In contrast, no increases in xeruborbactam MIC values were observed for these strains (Table S5). Based on binding assays using K. pneumoniae membrane preparations, xeruborbactam interacts with BPP2 and PBP3 with similar affinities, consistent with observations that mutations in individual PBPs did not affect the xeruborbactam MIC (Table 4).

To gain additional insight into the mode of antibacterial effects of xeruborbactam, we attempted to isolate single-step mutants with increased xeruborbactam MICs. No colonies appeared on agar plates containing xeruborbactam at ≥2× MIC after plating ~5 × 10^8^ CFU/mL of E. coli strain ECM5497 or A. baumannii strain AB1007 and incubating the plates for 48 h at 37°C (frequency of resistance [FOR], <2 × 10^−9^). The frequency of resistance for K. pneumoniae strain KPM1026a at 4× MIC was also low, <3 × 10^−9^. However, mutants were selected from this strain at 2× MIC of xeruborbactam with a FOR of 2 × 10^−8^. Several studied mutants had similar susceptibility profiles: a 2-fold increase in the xeruborbactam MIC value (from 16 μg/mL to 32 μg/mL) and 2-fold increases in the MIC values for several beta-lactam antibiotics, including the monobactams aztreonam, cephalosporin, and cefepime and the carbapenem meropenem. Much higher increases in MICs were observed for amdinocillin and the DBO BLIs avibactam, zidebactam, and durlobactam, which are all specific inhibitors of PBP2 only (Table 5) (6, 7, 9, 10, 37). Two mutants, strains KPM3532 and KPM3534, were studied using whole-genome sequencing, and neither contained mutations in any of the PBPs. As was discussed above, xeruborbactam appears to inhibit several essential PBPs with similar affinities in K. pneumoniae (Table 4). Interestingly, both K. pneumoniae mutants caried amino acid substitutions in the protein TolQ, M189K and S31F. Targeted sequencing of the *tolQ* gene in five more mutants identified various and nonidentical mutations in all of them (Table 5). *tolQ* is part of the *tol-pal* operon, with the TolQ protein as one of the three proteins localized to the cytoplasmic membrane that are part of the Tol-Pal transenvelope complex. The Tol-Pal complex promotes outer membrane constriction during cell division, coordinates the restructuring of peptidoglycan at division sites, and stabilizes the connection between the outer membrane and the underlying cell wall (38, 39). Inactivation of the gene *tolB* that encodes the periplasmic component of the Tol-Pal complex was discovered earlier in one of the mutants selected for resistance to nacubactam in E. coli (40), indicating that inactivation of Tol-Pal can compensate for the inhibition of PBP2. The observed cross-resistance between xeruborbactam (albeit at a low level) and PBP2 inhibitors is consistent with PBP2 being at least one of the xeruborbactam targets. Considering that *tolQ* is a relatively small gene (681 bp) and the whole *tol-pal* operon is ~5 kb, it might be of significance that no mutations conferring low-level xeruborbactam resistance in other *tol* genes were identified in KPM1026a. It raises the intriguing possibility that xeruborbactam’s mode of action might involve cell wall biogenesis targets (e.g., TolQ) in addition to the essential PBPs. While more studies are required to elucidate the exact mechanism of xeruborbactam’s antibacterial activity more completely, overall, the results of resistance studies indicate that xeruborbactam does inhibit specific steps in cell wall biogenesis.

**TABLE 5:** Antibiotic susceptibility profile of mutants with increased xeruborbactam MICs

Strain	Description	MIC (μg/mL) of^*a*^:							
		Xeruborbactam	Avibactam	Zidebactam	Durlobactam	Mecillinam	Meropenem	Cefepime	Aztreonam
KPM1026a	Parent	16	16	0.25	0.25	0.25	0.03	0.03	0.06
KPM3532	TolQ::M189K	32	>64	>64	8	>64	0.06	0.06	0.06
KPM3534	TolQ::S31F	32	>64	>64	16	>64	0.06	0.125	0.125
KPM4235	TolQ::IP(183–1844)PR	32	>64	>64	16	>64	0.06	0.125	ND
KPM4236	TolQ::TAA at aa 103^*b*^	32	>64	>64	16	>64	0.06	0.125	ND
KPM4238	TolQ::S25F	32	>64	>64	16	>64	0.06	0.125	ND
KPM4239	TolQ::S28F	32	>64	>64	16	>64	0.06	0.125	ND
KPM4240	TolQ::S25Y	32	>64	>64	16	>64	0.06	0.125	ND

a ND, not determined.

b aa, amino acid.

### Mutations conferring low-level resistance to the antibacterial activity of xeruborbactam have little or no effect on antibiotic potentiation by xeruborbactam through either beta-lactamase or PBP inhibition.

In order to evaluate the effect of the *tolQ* mutations on antibiotic potentiation by xeruborbactam through beta-lactamase inhibition, KPC- and NDM-containing plasmids were introduced into two mutants, strains KPM3532 and KPM3534, by conjugation. The meropenem or cefepime MICs in the presence of 4 μg/mL or 8 μg/mL of xeruborbactam against the resulting strains were compared with the MIC against the KPC- or NDM-containing parent strain (KPM1026a). Xeruborbactam increased the potency of meropenem or cefepime against the mutant derivatives to an extent similar to that observed in KPM1026a derivatives, indicating that *tolQ* mutations that confer low-level resistance to xeruborbactam alone do not affect its ability to enhance antibiotic potency due to inhibition of target beta-lactamases (Table 6).

**TABLE 6:** Effects of mutations causing increased xeruborbactam MICs on enhancement of antibiotic potency by xeruborbactam through KPC or NDM inhibition

Strain	Description	MIC (μg/mL) of:						
		Xeruborbactam	Meropenem			Cefepime		
			Alone	With xeruborbactam at:		Alone	With xeruborbactam at:	
				4 μg/mL	8 μg/mL		4 μg/mL	8 μg/mL
KPM1271	KPM1026a/KPC-3^*a*^	16	16	≤0.06	≤0.06	4	≤0.06	≤0.06
KPM4127	KPM3532 (TolQ::M189K)/KPC-3	32	32	0.125	≤0.06	8	≤0.06	≤0.06
KPM4129	KPM3532 (TolQ::M189K)/NDM-1	32	>64	0.25	0.125	8	≤0.06	≤0.06
KPM1281	KPM1026a/NDM-1^*a*^	16	>64	0.125	≤0.06	16	≤0.06	≤0.06
KPM4128	KPM3534 (TolQ::S31F)/KPC-3	32	32	0.125	≤0.06	32	0.25	0.125
KPM4130	KPM3534 (TolQ::S31F)/NDM-1	32	>64	0.25	0.125	32	0.25	0.125

a KPC-3 and NDM-1 plasmids were introduced into KPM1026a and its mutants by conjugation using K. pneumoniae KP1074 and E. coli EC1061 as the donor, respectively (see Materials and Methods).

Multiple studies demonstrated that beta-lactamase inhibitors that also inhibit PBP2, such as nacubactam, zidebactam, and durlobactam, might enhance the potency of beta-lactam antibiotics in the absence of beta-lactamases (7, 9, 41, 42). This enhancement effect can also be seen for xeruborbactam, presumably due to inhibition of multiple PBPs. Reductions in MICs by 2- to 8-fold for multiple beta-lactams in the presence of xeruborbactam (2 to 8 μg/mL) were observed for beta-lactamase-negative strains K. pneumoniae KPM1026a, A. baumannii AB1007, and P. aeruginosa PAM1154 (Table S7). In contrast, no reduction of the beta-lactam MIC against KPM1026a was seen in the presence of vaborbactam (up to 64 μg/mL), an agent that does not have antibacterial activity (data not shown).

We investigated the effects of the *tolQ* mutations in the KPM1026a background on antibiotic potentiation by xeruborbactam through PBP inhibition. The mutations had negligible effects on the enhancement of the potency of meropenem or cefepime by xeruborbactam: 2-fold decreases in MICs with xeruborbactam at 4 μg/mL in both the KPM1026a and *tolQ* mutants and 8- to 16-fold increases in MICs in KPM1026a versus 4- to 8-fold increases in MIC in the *tolQ* mutants with xeruborbactam at 8 μg/mL. Somewhat less potentiation by xeruborbactam in the *tolQ* mutants compared to that in KPM1026a was observed for aztreonam and ceftibuten. At the same time, xeruborbactam completely reversed the *tolQ*-mediated resistance to the PBP2 inhibitors, such as amdinocillin or the DBO BLIs zidebactam and durlobactam (Table 7). Hence, enhancement of the potency of various PBP inhibitors by xeruborbactam is mostly retained even when its antibacterial activity is reduced. Of note, previous studies demonstrated that multiple mutations that confer resistance to the antibacterial activity of DBO BLIs also do not affect their beta-lactam enhancement properties (9, 43).

**TABLE 7:** Effects of mutations causing increased xeruborbactam MICs on enhancement of antibiotic potency by xeruborbactam through PBP inhibition

Antibiotic	MIC (μg/mL) (fold decrease compared to MIC of antibiotic alone)								
	KPM1026a			KPM3532 (TolQ::M189K)			KPM3534 (TolQ::S31F)		
	Alone	With xeruborbactam at:		Alone	With xeruborbactam at:		Alone	With xeruborbactam at:	
		4 μg/mL	8 μg/mL		4 μg/mL	8 μg/mL		4 μg/mL	8 μg/mL
Meropenem	0.03	0.015 (2)	0.004 (8)	0.06	0.03 (2)	0.015 (4)	0.06	0.03 (2)	0.015 (4)
Cefepime	0.03	0.015 (2)	0.002 (16)	0.06	0.03 (2)	0.015 (4)	0.125	0.03 (4)	0.015 (8)
Aztreonam	0.06	0.015 (4)	0.004 (16)	0.06	0.06 (1)	0.06 (1)	0.125	0.03 (4)	0.03 (4)
Ceftibuten	0.06	0.015 (4)	0.008 (8)	0.125	0.06 (2)	0.06 (2)	0.125	0.06 (2)	0.03 (4)
Mecillinam	0.25	≤0.06	≤0.06	>64	0.5	0.5	>64	0.5	0.5
Zidebactam	0.25	≤0.06	≤0.06	>64	0.25	0.125	>64	0.25	0.125
Durlobactam	0.25	0.125	≤0.06	8	0.25	0.125	16	0.25	0.125
Xeruborbactam	16	NA^*a*^	NA	32	NA	NA	32	NA	NA

a NA, not applicable.

### Potent broad-spectrum beta-lactamase inhibition by xeruborbactam combined with its intrinsic antibacterial activity provides added benefit to xeruborbactam/beta-lactam combinations.

We investigated the effects of two agents with differences in direct antibacterial activity, xeruborbactam and vaborbactam, on meropenem MICs against KPC-producing strains using either the panel of isogenic engineered strains producing KPC-3 or the panel of clinical KPC-producing strains and their derivatives that lost KPC plasmids. Vaborbactam at 64 μg/mL reduced the meropenem MIC values to the levels observed in the absence of KPC, including in strains with multiple porin mutations and increased efflux (Tables 8 and 9). Testing xeruborbactam in combination with meropenem against the panel of isogenic porin/efflux mutants producing KPC-3 demonstrated that 1 μg/mL of xeruborbactam was sufficient to reverse meropenem resistance to the levels observed in the respective strains that lacked the KPC-3 enzyme for the strains that had AcrAB overexpressed or OmpK35 or OmpK36 inactivated. Xeruborbactam at 8 μg/mL decreased the meropenem MIC values for the same strains to levels 8- to 16-fold lower than the MIC values for the respective KPC-3-lacking strains. For the mutants that either lacked both OmpK35 and OmpK36 (strain KPM2631) or had increased efflux in addition to defective porins (strain KPM1273), 8 μg/mL of xeruborbactam reduced the meropenem MIC values to levels 2-fold lower than the MIC values for the respective KPC-3-lacking strains (Table 8). Against several KPC-producing clinical strains, the MIC values of meropenem in combination with xeruborbactam at 8 μg/mL were also 2- to 8-fold lower than the MIC values for meropenem alone against the respective KPC-lacking strains (a proxy for the complete inhibition of KPC) (Table 9).

**TABLE 8:** Potency of xeruborbactam and vaborbactam to enhance the activity of meropenem against the isogenic strains of KPC-3-producing K. pneumoniae with various combinations of efflux and porin mutations

KPC-3-containing strain	No-plasmid strain	Construction^*a*^	Description of^*b*^:			Meropenem MIC (μg/mL) with KPC-3 alone or with indicated BLI					MIC (μg/mL)		
			OmpK35	OmpK36	AcrAB	Alone	With xeruborbactam at:		With vaborbactam at:				
							1 μg/mL	8 μg/mL	8 μg/mL	64 μg/mL	No-plasmid strain	Xeruborbactam^*c*^	Vaborbactam
KPM1271	KPM1026a	Wild type	FL	FL	BL	16	0.03	0.004	0.03	0.03	0.03	16	>128
KPM1272	KPM1027	*ramR*_fs	Down	FL	Up	16	0.03	0.004	0.06	0.06	0.06	16	>128
KPM2601	KPM2600	Δ*ompK35*	NF	FL	BL	32	0.03	0.008	0.06	0.06	0.06	16	>128
KPM2067	KPM2040	*ompK36*_fs	FL	NF	BL	64	0.125	0.008	0.125	0.125	0.125	16	>128
KPM2631	KPM2613	*ompK36*_fs Δ*ompK35*	NF	NF	NL	256	1	0.125	1	0.25	0.25	32	>128
KPM1273	KPM1171	*ramR*_fs *ompK36_1171*	Down	NF	Up	>256	8	1	16	2	2	64	>128

a *ramR*_fs and *ompK36*_fs encode truncated proteins due to frameshift mutations.

b FL, full length; NF, nonfunctional; BL, basal level; Up, overexpressed; Down, downregulated.

c Xeruborbactam MICs against strains producing and lacking KPC-3 were the same.

**TABLE 9:** Potency of meropenem alone or in combination with xeruborbactam or vaborbactam against selected KPC-producing strains and their plasmid-loss derivatives

KPC-producing strain	Beta-lactamases	Description of^*a*^:		Meropenem MIC (μg/mL)			KPC-lacking derivative strain	Beta-lactamase(s)	Meropenem MIC (μg/mL)		
		OmpK35	OmpK36	Alone	With xeruborbactam at 8 μg/mL	With vaborbactam at 64 μg/mL			Alone	With xeruborbactam at 8 μg/mL	With vaborbactam at 64 μg/mL
KP1004	KPC-2, TEM-1, SHV-11	FS from aa 42	Full copy	16	0.004	0.03	KPM1206	SHV-11	0.03	0.008	0.03
KP1074	KPC-3, SHV-11, TEM	FS from aa 42	GD	64	0.03	0.125	KPM1211	SHV-11	0.125	0.06	0.125
KP1084	KPC-3, SHV-11, TEM-1	FS from aa 42	GD	64	0.06	0.125	KPM1311	SHV-11	0.125	0.03	0.125
KP1087	KPC-2, CTX-M-15, SHV-11, TEM-1	FS from aa 208	GD	64	0.06	0.125	KPM1304	SHV-11 CTX-M-15	1	0.06	0.125
KP1093	KPC-3, SHV-11, TEM-1	FS from aa 42	GD	128	0.125	0.25	KPM2042	SHV-11	0.25	0.06	0.125
KP1094	KPC-2, TEM-1, LEN-17	Stop at aa 230	Stop at aa 92	>256	0.25	0.25	KPM1977	LEN-17	0.5	0.25	0.25
KP1100	KPC-3, TEM, SHV	FS from aa 42	GD	>256	0.125	0.5	KPM2136	SHV-11	0.5	0.03	0.25
KP1251	KPC-3, TEM, SHV	FS from aa 42	Full copy	64	0.004	0.03	KPM2626	SHV	0.03	0.004	0.03
KP1254	KPC-2, TEM, SHV, OXA-10	FS from aa 42	IS insertion and deletion	>256	2	4	KPM2841	SHV-11 OXA-10	4	1	2
KP1099	KPC-2, SHV-11, CTX-M-14, SHV-12	FS from aa 29	GD	128	0.125	0.25	KPM2898	SHV-11 CTX-M-14	2	0.06	0.25

a aa, amino acid; FS, frameshift; GD, insertion of two amino acids, Gly134Asp135, in the L3 loop of OmpK36, resulting in a partially functional OmpK36 with a constricted channel.

The results described above translated well to the panel of 141 KPC-producing clinical isolates of K. pneumoniae that were enriched in strains with mutations in OmpK36. The MIC_90_ of meropenem in combination with vaborbactam at 64 μg/mL (assumes complete reversal of KPC-mediated meropenem resistance even in the presence of porin mutations) was the same as with xeruborbactam at 4 μg/mL (Table 10). With xeruborbactam at 8 μg/mL, the meropenem MIC_90_ was reduced 4-fold; the same 4-fold reduction of MIC_90_ was observed for the subset of 92 strains with nonfunctional OmpK36. This increased extent of meropenem’s potentiation of xeruborbactam compared to its potentiation of vaborbactam is consistent with additional synergy due to PBP inhibition by both meropenem and xeruborbactam under the conditions of inhibition of beta-lactamases by xeruborbactam.

**TABLE 10:** *In vitro* potency of xeruborbactam and vaborbactam to enhance the activity of meropenem against the clinical strains of KPC-producing K. pneumoniae according to the functional status of OmpK36

Strain, measure of potency	Meropenem MIC (μg/mL)					MIC (μg/mL) of:	
	Alone	With vaborbactam at:		With xeruborbactam at:			
		8 μg/mL	64 μg/mL	4 μg/mL	8 μg/mL	Vaborbactam	Xeruborbactam
All strains (*n* = 141)							
MIC_50_	128	1	0.125	0.125	≤0.06	>64	>16
MIC_90_	256	16	1	1	0.25	>64	>16
Strains with							
Functional OmpK36							
MIC_50_	16	0.03	0.03	≤0.06	≤0.06	>64	16
MIC_90_	128	0.06	0.06	≤0.06	≤0.06	>64	>16
Nonfunctional OmpK36							
MIC_50_	256	2	0.25	0.25	0.125	>64	>16
MIC_90_	256	16	2	2	0.5	>64	>16

The MICs of several other beta-lactams (ceftazidime, cefepime, and aztreonam) for the KPC-3-producing K. pneumoniae strain KP1074 (sequence type 512 [ST512], with OmpK35 inactivated and a partially functional OmpK36) measured in the presence of 8 μg/mL xeruborbactam were also 2- to 8-fold below the values that correspond to complete inhibition of KPC (Table S8). Given pharmacokinetics and pharmacodynamics (PK-PD) considerations, correlations with meropenem MICs in combination with xeruborbactam at 8 μg/mL, and *in vivo* bactericidal effects, 8 μg/mL is the expected fixed concentration of xeruborbactam for susceptibility testing. The data indicate that at this concentration, in addition to the broad-spectrum beta-lactamase inhibition, xeruborbactam may exert an additional enhancement through direct antimicrobial activity, which would be most pronounced in strains with xeruborbactam MICs of ≤32 μg/mL.

### Conclusions.

Xeruborbactam is a broad-spectrum inhibitor of numerous serine and metallo-beta-lactamases with a spectrum of inhibition of enzymes that includes activity in Acinetobacter spp., Pseudomonas aeruginosa, and *Enterobacterales* (13). In addition to this activity, xeruborbactam also has direct antibacterial activity against some Gram-negative and Gram-positive bacteria that is generally above the concentrations associated with beta-lactamase inhibition, and it also enhances the potency of multiple beta-lactam antibiotics even in strains that lack beta-lactamase enzymes. Xeruborbactam binds to PBPs, and it also induces changes of cellular morphology comparable with the effect of meropenem (at least for K. pneumoniae and P. aeruginosa). Thus, the mode of antibacterial activity of xeruborbactam is consistent with inhibiting the function of multiple PBPs. Mutations conferring low-level resistance to the antibacterial effects of xeruborbactam localized to the gene *tolQ* are also consistent with its targeting cell wall biogenesis and point to targets in addition to essential PBPs. Importantly, these mutations do not affect antibiotic potentiation by xeruborbactam due to inhibition of either beta-lactamases or essential PBPs. The intrinsic activity of xeruborbactam might provide added benefit to xeruborbactam combinations with multiple beta-lactam antibiotics compared to BLIs that lack direct antibacterial activity.

## MATERIALS AND METHODS

### Strains.

(i) Panels of engineered bacterial strains containing various combinations of porin and efflux mutations were used. The construction of all the strains used in the study is described in detail in references 23, 44, and 45. A detailed description of all strains used in the study is provided in Table S1. (ii) KPC-producing clinical isolates and their derivatives that lost KPC plasmids were used also. These strains and their origins are described in the Table S2. (iii) Finally, KPC-producing clinical isolates enriched in strains with porin mutations were used. The mutations identified include various deletions, insertions, nonsense mutations, and decreased expression. Most of the isolates were obtained from JMI Laboratories or International Health Management Associates (IHMA); they were collected in the course of global surveillance studies.

### Antimicrobial susceptibility testing.

Bacterial isolates were subjected to broth microdilution susceptibility testing, performed according to Clinical and Laboratory Standards Institute (CLSI) methods (46), using panels prepared in-house. Meropenem was purchased from Sandoz, and all other antibiotics were from Sigma-Aldrich. Xeruborbactam was synthesized at Qpex Biopharma, San Diego, CA. Avibactam was purchased from eNovation Chemicals LLC, Bridgewater, NJ, USA; zidebactam was from MedKoo Biosciences, Inc., Morrisville, NC, and nacubactam was from Advanced ChemBlocks, Hayward, CA. Durlobactam was synthesized at Acme Bioscience, Palo Alto, CA, USA.

### Conjugation in K. pneumoniae.

Conjugation was used to construct strain KPM1026a, KPM3532, and KPM3534 derivatives that carried plasmids with *bla*_KPC-3_ or *bla*_NDM-1_ genes. Conjugation was performed as previously described (45) with modifications. First, streptomycin-resistant mutants of recipient strains were isolated on LB (Luria-Bertani) agar containing streptomycin at 40 μg/mL. Next, both donor and recipient strains were grown overnight in LB broth at 37°C with aeration. On the next day, the donor and recipient cultures (100 μL each) were mixed and pelleted by centrifugation for 1 min at room temperature. The cells were resuspended in 40 μL of LB broth and spotted onto an LB agar plate without antibiotics. Recipient-only and donor-only cultures were similarly spotted on LB agar plates as negative controls. The plates were incubated at 37°C for 4 to 5 h, and cells were collected and resuspended in 0.5 mL of LB medium to an optical density at 600 nm (OD_600_) of 0.1 to 0.5. Then, 0.05 to 0.1 mL of cell suspension was plated on a plate containing streptomycin and meropenem at 50 μg/mL and 2 μg/mL, respectively. Transconjugants were verified by PCR and DNA sequencing.

### Single-step mutant selection.

Beta-lactamase-negative strains of E. coli (strain ECM5497), A. baumannii (strain AB1007), and K. pneumoniae (strain KPM1026a) were used in resistance development studies. Approximately 5 × 10^8^ cells from fresh cultures (originating from a single colony) were transferred to agar plates containing xeruborbactam at 2× to 8× MIC. Colonies were incubated for 24 h at 37°C before the frequency of resistance emergence was calculated as the ratio of the number of CFU grown on the xeruborbactam-containing plate over the number of CFU recorded for xeruborbactam-free plates. Single colonies from xeruborbactam-containing plates were grown twice on a nonselective medium before their antimicrobial-resistance levels were assessed. For K. pneumoniae KPM1026a, several independent cultures were used in resistance studies.

### Preparation of total bacterial membrane proteins from Gram-negative bacteria.

Ten milliliters of overnight bacterial culture was inoculated in 1 L of LB medium and grown to an OD_600_ of 0.7 to 0.9. The cell pellet was washed once with 50 mL of solution containing 100 mM Na-phosphate at pH 7.0 and 100 mM NaCl and resuspended in 10 mL of the same buffer with one tablet of EDTA-free mini-protease inhibitor and 10 mg of lysozyme. Cell suspensions were incubated for 45 min at 37°C, and then 200 μL of DNase I was added along with MgSO_4_ (to a final concentration of 10 mM), followed by three cycles of freeze-thawing (10 min and 20 min, respectively). Four cycles of sonication at maximum power were performed next, each cycle lasting for 30 s with 1 min on ice between cycles. The cell suspension was centrifuged at 12,000 × *g* for 10 min at 4°C. The supernatant was collected and centrifuged at 100,000 × *g* for 45 min at 4°C. The pellet was washed three times with 10 mL of 100 mM Na-phosphate, pH 7.0, by resuspension and subsequent centrifugation at 100,000 × *g* for 10 min at 4°C. The pellet was finally resuspended in 1.5 mL of 100 mM Na-phosphate, pH 7.0, and passed several times through a Dounce homogenizer (tight pestle). Membrane preparations were aliquoted and kept at −80°C.

### *In vitro* PBP binding assay.

Five microliters of total bacterial membrane preparation was mixed with 5 μL of a test compound at a desired concentration in 100 mM Na-phosphate buffer, pH 7.0. Incubation of bacterial membrane preparations with control beta-lactams was performed for 10 min at 30°C; xeruborbactam was incubated with membrane preparations for 1 h at 30°C. Five microliters of 150 μM Bocillin FL fluorescent probe (Thermo Fisher Scientific) was added, and the reaction mixture was incubated for an additional 10 min at 30°C in the case of beta-lactams or for 20 min on ice (0°C) in the case of xeruborbactam. We believe that the 0°C temperature is required to prevent displacement of xeruborbactam from its complex by the Bocillin probe. At the end of the incubation step, 15 μL of 2× SDS sample loading buffer was added, and the reaction mixture was incubated for 5 min at 95°C. Twenty-five microliters of the reaction mixture was then loaded on an 8% SDS Tris-glycine gel (Thermo Fisher Scientific). At the end of the run, the gel was briefly rinsed with water and fixed in 10% acetic acid, 40% methanol for 20 min. After an additional wash in water for 30 min, the gel was scanned on a Typhoon Trio+ Imager using a blue laser filter (excitation, 488 nm, and emission, 520 nm). Densitometry of the gel images was performed using ImageQuant software (Molecular Dynamics). IC_50_ values were calculated with Prism software (GraphPad).

### Fluorescence microscopy.

Single colonies of K. pneumoniae strain KPM1026a, A. baumannii strain ATCC 17978, and P. aeruginosa strain PAM1154 were picked from LB agar plates, inoculated in cation-adjusted Mueller-Hinton broth (CA-MHB), and incubated overnight at 30°C with rolling. Overnight cultures were diluted into fresh CA-MHB and incubated at 30°C, with rolling, until they reached an OD_600_ of ≈0.12 to 0.15 (equivalent to ~2 × 10^8^ CFU/mL). Samples were then treated with compounds and incubated at 30°C, with rolling. After 1 or 2 h of treatment, cells were collected and stained with a dye mix containing FM4-64 (1 μg/mL), DAPI (4′,6-diamidino-2-phenylindole) (2 μg/mL), and SYTOX green (0.5 μM) (Molecular Probes, Invitrogen, or Thermo Fisher Scientific). Cells were transferred onto an agarose pad containing 1.2% agarose and 20% LB medium for microscopy. Microscopy was performed as previously described (34).
