# Supplementary material for: Intrinsic Antibacterial Activity of Xeruborbactam In Vitro: Assessing Spectrum and Mode of Action
Source: Antimicrob Agents Chemother. 2022 Sep 14;66(10):e00879-22. doi: 10.1128/aac.00879-22 (PMC9578396; doi:10.1128/aac.00879-22)
Supplement: Supplemental file 1 — Supplemental material. Download aac.00879-22-s0001.pdf, PDF file, 0.7 MB [file aac.00879-22-s0001.pdf]

Supplementary Tables

Table S1. Laboratory strains used in this study

Strains	Acquired Beta-lactamase	Parent strain/ Recipient	Relevant genotype	Reference
Klebsiella pneumoniae				
Isogenic panel[1]				
KPM1026a	none	ATCC 43816	Wild type	(1)
KPM1271	KPC-3[2], TEM-1	KPM1026a	Wild type	(1)
KPM1281	NDM-1	KPM1026a	Wild type	This study
KPM3532	none	KPM1026a	*tolQ::M189K*	This study
KPM4127	KPC-3, TEM-1	KPM3532	*tolQ::M189K*	This study
KPM4129	NDM-1[3]	KPM3532	*tolQ::M189K*	This study
KPM3534	none	KPM1026a	*tolQ::S31F*	This study
KPM4128	KPC-3, TEM-1	KPM3534	*tolQ::S31F*	This study
KPM4130	NDM-1	KPM3534	*tolQ::S31F*	This study
KPM1027	none	KPM1004	*ramR_fs*[4]_aa46	(1)
KPM1272	KPC-3, TEM-1	KPM1027	*ramR_fs*_aa46	(1)
KPM2040	none	KPM2067	*ompK36_fs_aa54*	(1)
KPM2067	KPC-3, TEM-1	KPM1271	*ompK36_fs_aa54*	(1)
KPM2600	none	KPM1026a	*ΔompK35*	(1)
KPM2601	KPC-3, TEM-1	KPM2600	*ΔompK35*	(1)
KPM2613	none	KPM2040	*ompK36_fs_aa54 ΔompK35*	(1)
KPM2631	KPC-3, TEM-1	KPM2613	*ompK36_fs_aa54 ΔompK35*	(1)
KPM1171	none	KPM1027	*ramR_fs*_aa46 *ompK36_del48aa_aa318*	(1)
KPM1273	KPC-3, TEM-1	KPM1171	*ramR_fs*_aa46 *ompK36_del48aa_aa318*	(1)
Pseudomonas aeruginosa isogenic efflux panel[5]				
PAM1020	none	PA01	*Wild type*	(2)
PAM1154	none	PAM1020	*oprM::Hg*	(2)
PAM1106	none	PAM1020	*mexA::Tet*	(2)
PAM1032	none	PAM1020	*mexR:: L75R*	(2)
PAM1033	none	PAM1020	*nfxB :: T39I*	(2)
PAM1034	none	PAM1020	*mexT::R242P*	(2) (19)
Acinetobacter baumannii panel[5]				
AB1007	none	Clinical isolate	*Wild-type*	(3)
ACM1027	none	AB1007	*adeN_fs_aa156*	(3)
ACM1030	none	AB1007	*adeS::P154T*	(3)
ACM1013	none	ACM1010	*adeR ΔadeIJK*	(4)
ACM1014	none	ACM1010	*ΔadeABC*	(4)
ACM1015	none	ACM1010	*ΔadeIJK ΔadeABC*	(4)

[1]All isogenic strains of *K. pneumoniae* are derivatives of KPM1026a which is a streptomycin resistant mutant of ATCC of ATCC43816.

[2]All KPC-3-producing strains were constructed by conjugating the plasmid pKpQIL from the clinical isolate of *K. pneumoniae* KP1074 (ATCC BAA-2814) into various isogenic derivatives of KPM1026a. The *bla*$_{TEM-1}$ gene is located on pKpQIL plasmid together with *bla*$_{KPC-3}$.
[3]The NDM-1 producing strains were constructed by conjugating a plasmid that carry the bla$_{NDM-1}$ gene from the clinical isolate of *E. coli* EC1061 into KPM1026a and its mutants.
[4] fs_aa#XX, frame-shift at amino acid no. XX, that results in non-functional protein
[6] Isogenic strains of P. aeruginosa are derivatives of PAM1020 (PA01).
[6] Isogenic strains of *A. baumannii* are derivatives of AB1007, antibiotic susceptible clinical isolates. ACM1027 and ACM1030 are *adeN* and *adeS* mutants, respectively, that were selected from AB1007 on tigecycline at 0.5 µg/ml.

Table S2. Selected KPC-producing strains and their "plasmid-loss" derivatives

KPC-producing strain	Beta-lactamases	OmpK35 protein[1]	OmpK36 protein[2]	KPC-lacking derivative strain	Beta-lactamases
**KP1004**	KPC-2, TEM-1, SHV-11	FS from aa #42	Full copy	**KPM1206**[3]	SHV-11
**KP1074**	KPC-3, SHV-11, TEM	FS from aa #42	GD	**KPM1211**[3]	SHV-11
**KP1084**	KPC-3, SHV-11, TEM-1	FS from aa #42	GD	**KPM1311**[4]	SHV-11
**KP1087**	KPC-2, CTX-M-15, SHV-11, TEM-1	FS from aa #208	GD	**KPM1304**[4]	SHV-11 CTX-M-15
**KP1093**	KPC-3, SHV-11, TEM-1	FS from aa #42	GD	**KPM2042**[4]	SHV-11
**KP1094**	KPC-2 TEM-1 LEN-17	Stop at aa #230	Stop at aa #92	**KPM1977**[3]	LEN-17
**KP1100**	KPC-3 TEM SHV	FS from aa #42	GD	**KPM2136**[3]	SHV-11
**KP1251**	KPC-3 TEM SHV	FS from aa #42	Full copy	**KPM2626**[5]	SHV
**KP1254**	KPC-2 TEM SHV OXA-10	FS from aa #42	IS insertion and deletion	**KPM2841**[5]	SHV-11 OXA-10
**KP1099**	KPC-2 SHV-1 1CTX-M-14 SHV-12	FS from aa #29	GD	**KPM2898**[6]	SHV-11 CTX-M-14

[1]OmpK35 protein: various frame-shift (FS) and non-sense (stop) mutations resulted in generation of a truncated, non-functional protein.
[2]OmpK36 protein: GD, duplication of two amino acids, Gly134 (G) and Asp135 (D), located in loop 3 of OmpK36, results in a constricted pore and has been shown to be associated with meropenem resistance
[3] KPC-containing plasmid was lost after the parental strain was treated with the humanized exposure of meropenem-vaborbactam in in vitro PK-PD Hollow-Fiber studies.
[4] KPC-containing plasmid was lost after the parental strain was treated with a combination of 100 µg/ml meropenem and 100 µg/ml vaborbactam for up to 18 hours

[5] KPC-containing plasmid was lost after multiple passages under non-selective conditions (MHA)
[6] First, the strain KPM2895 was selected on meropenem-vaborbactam from KP1099 as a single-step mutant with reduced susceptibility to meropenem-vaborbactam.; it was shown to have an increased copy number of the KPC-containing plasmid as compared to KP1099. This also led to the apparent plasmid instability.

Table S3. In vitro activity of xeruborbactam against selected strains of gram-positive bacteria

		MIC (µg/ml)		
**Strain**	**Organism**	**Xeruborbactam**	**Meropenem**	**Levofloxacin**
ATCC 29213	MSSA	4	0.06	0.125
sa002	MSSA	8	0.125	0.125
sa003	MSSA	8	0.125	0.125
sa004	MSSA	8	0.125	0.125
sa005	MSSA	8	0.125	0.125
sa006	MSSA	8	0.125	0.06
sa007	MSSA	8	0.25	0.125
sa008	MSSA	8	0.125	0.25
sa009	MSSA	8	0.125	0.125
sa011	MSSA	8	0.125	0.25
sa012	MSSA	8	0.125	0.25
sa015	MSSA	8	0.25	0.125
sa001	MRSA	>32	16	8
sa010	MRSA	>32	32	8
sa013	MRSA	>32	16	0.25
sa014	MRSA	>32	8	0.125
sa148	MRSA	>32	32	16
sa150	MRSA	>32	32	8
sa151	MRSA	>32	32	16
sa152	MRSA	>32	4	0.125
sa153	MRSA	>32	32	16
sa278	MRSA	>32	32	32
sa279	MRSA	>32	4	2
sa280	MRSA	>32	4	2
sa281	MRSA	>32	32	32
sp001	*Streptococcus pneumoniae*	8	≤0.03	0.5
sp002	*Streptococcus pneumoniae*	4	≤0.03	0.5
sp003	*Streptococcus pneumoniae*	4	≤0.03	1
sp004	*Streptococcus pneumoniae*	>32	0.25	1
sp005	*Streptococcus pneumoniae*	16	0.06	0.5
sp006	*Streptococcus pneumoniae*	32	0.25	0.5

sp007	*Streptococcus pneumoniae*	16	0.125	1
sp008	*Streptococcus pneumoniae*	32	0.125	0.5
sp009	*Streptococcus pneumoniae*	32	0.25	1
sp117	*Streptococcus pneumoniae*	>32	0.25	2
sp118	*Streptococcus pneumoniae*	>32	0.25	1
sp119	*Streptococcus pneumoniae*	32	0.25	1
sp120	*Streptococcus pneumoniae*	32	0.25	1
sp121	*Streptococcus pneumoniae*	32	0.125	1
sp122	*Streptococcus pneumoniae*	>32	4	32
sp123	*Streptococcus pneumoniae*	>32	0.25	0.5
sp125	*Streptococcus pneumoniae*	32	0.25	1
sp126	*Streptococcus pneumoniae*	>32	0.25	1
efs003	*Enterococcus faecalis*	>32	2	0.5
efs004	*Enterococcus faecalis*	>32	4	1
efs005	*Enterococcus faecalis*	>32	4	1
efs006	*Enterococcus faecalis*	>32	2	1
efs007	*Enterococcus faecalis*	>32	4	1
efs026	*Enterococcus faecalis*	>32	2	0.5
efs071	*Enterococcus faecalis*	>32	4	32
efs072	*Enterococcus faecalis*	>32	2	16
efs073	*Enterococcus faecalis*	>32	4	1
efs090	*Enterococcus faecalis*	>32	1	32
efs091	*Enterococcus faecalis*	>32	1	32
efs092	*Enterococcus faecalis*	>32	8	32
efs093	*Enterococcus faecalis*	>32	16	16
efs094	*Enterococcus faecalis*	>32	>32	32
efs095	*Enterococcus faecalis*	>32	4	2
efs096	*Enterococcus faecalis*	>32	4	32

MSSA, methicillin-susceptible *Staphylococcus aureus*; MRSA, methicillin-resistant *Staphylococcus aureus*

Table S4. Assessment of the impact of *ompK35* and *ompK36* mutations on xeruborbactam MIC values in clinical isolates of *K. pneumoniae* (N=453)

			OmpK36		
			Full Length	GD	Non-functional
			228 (5.3%)	160 (75%)	65 (92.3%)
OmpK35	Full-length	118 (31.4%)	82 (6.1%)	11 (82%)	25 (92%)
	Non-functional	318 (49%)	130 (3.4%)	149 (74.5)	39 (95%)

Numbers in brackets indicate the percentage of strains with xeruborbactam MIC values ≥ 32 µg/ml

Percentage of strains with xeruborbactam MIC values ≥32 µg/ml were calculated for the groups of isolates stratified by the functional status of OmpK35 and OmpK36. OmpK35 and OmpK36 were considered as non-functional if the insertions, deletions, frame-shift or nonsense mutations were identified in the respective genes or if the significant reduction in the gene expression was detected. OmpK36 GD is a partially functional protein with a constricted channel due to the insertion of two amino amino acids, Gly134Asp135, into L3-loop.

Table S5. Assessment of the impact of PBP2 and PBP3 mutations on xeruborbactam MIC values in clinical isolates of *Escherichia coli*

Strain	Beta-lactamases	OmpK35	OmpK35 Functional Status	OmpK36	OmpK36 Functional Status	PBP2[1]	PBP3[2]	XER	DUR	ZID	ATM	ATM-AVI
EC1081	**CTX-M-15, OXA-1**	Full length	**FN**	Full length	**FN**	same as MG1655	Q227H	8	0.25	0.125	64	≤0.06
EC1085	**none**	Full length	**FN**	Full length	**FN**	**T331P**	A233T I332V	8	**32**[3]	**16**	2	0.125
EC1121	**CMY-6, CTX-M-15, NDM-1, OXA-2, TEM-1B-like**	FS from aa#31	**NF**	Full length	**FN**	V217M	**YRIN ins**, E349K, I532L	8	0.5	0.5	>64	**4**
EC1088	**KPC-3, OXA-9, TEM-1A**	Full length	**FN**	Full length	**FN**	A308G	same as MG1655	8	0.5	0.25	>64	0.125
EC1097	**KPC-3, TEM-1B**	Full length	**FN**	Full length	**FN**	**M574I**	Q227H	8	**1**	0.25	>64	0.5
EC1098	**CMY-6, NDM-1, OXA-2, TEM-1A-like**	FS from aa#31	**NF**	Full length	**FN**	V217M	**YRIN ins**, I532L	8	0.5	0.25	64	**4**
EC1103	**CMY-42, NDM-5, TEM-1**	Full length	**FN**	Full length	**FN**	same as MG1655	**YRIN ins**, Q227H, E349K, I532L	8	0.25	0.25	32	**8**
EC1105	**CTX-M-15, NDM-7, TEM-1B**	Full length	**FN**	Full length	**FN**	**A543T**	**YRIN ins**, Q227H, E349K, I532L	8	**1**	0.5	>64	**2**
EC1078	**CMY-2, CTX-M-14, TEM-1B**	FS from aa#251	**NF**	E130stop	**NF**	same as MG1655	I332V	32	8	8	64	1
EC1119	**CTX-M-15 OXA-1**	FS from aa#213	**NF**	IS1414 at nt#756	**NF**	same as MG1655	**YRIN ins**, Q227H, E349K, I532L	32	8	8	>64	**8**

Abbreviations: FN, functional; NF, non-functional; XER, xeruborbactam; DUR, durlobactam; ZID, zidebactam; ATM, aztreonam; AVI, avibactam.
[1]Amino acid substitutions in PBP2 associated with ≥4-fold increase in MIC to PBP2 inhibitors are in bold
[2]An insertion of four amino acids, YRIN, in PBP3 associated with the increase in MIC for PBP3 inhibitors are in bold letters. Aztreonam (a PBP3 inhibitor) was used in combination with avibactam as a control to unmask the impact of these insertions on clinical strains, many of which produced NDM (not inhibited by avibactam, hence no use for ceftazidime-avibactam as a control).
[3]MICs values that were increased ≥4-fold compared to that in the absence of PBP2 or PBP3 mutations are in bold numbers.

Table S6. Phenotypic assessment of the MexAB-OprM efflux pump functionality in the strains of *P. aeruginosa* with decreased xeruborbactam MIC values

Strain	Aztreonam	Aztreonam-Xeruborbactam (fixed 4)	Aztreonam-Xeruborbactam (fixed 8)	Xeruborbactam
1098084	0.25	0.25	0.125	16
1116835	0.5	≤0.03	ND[1]	8
1116849	2	0.25	0.25	16
1128636	2	2	2	32
1124145	0.25	0.25	0.125	16
1126521	0.5	0.25	ND	8
1119418	32	0.5	ND	8
1128674	4	0.25	0.125	16
1118234	32	8	0.25	16
1106973	2	0.06	≤0.03	16
1104168	1	0.5	0.5	32
1104284	0.5	0.25	ND	8
1130387	0.25	0.125	0.06	16
1114012	0.25	0.06	ND	8
1131123	0.25	0.06	ND	8
1097436	64	0.5	ND	8
1126957	>64	2	ND	8
1089003	0.5	0.5	0.5	16
1098916	0.5	0.25	0.25	32
1100407	0.25	0.125	0.125	32
1104100	0.12	≤0.03	≤0.03	16
1108885	0.25	0.125	≤0.03	16
1109744	0.5	0.125	0.06	16
1113238	32	0.125	ND	8
1113634	4	0.5	ND	8
1125954	1	0.5	0.5	32
1126982	0.5	0.25	0.125	32
1130475	0.12	≤0.03	≤0.03	16
1125996[2]	>64	64	4	32
1130447	>64	16	0.5	16

[1]ND, not determined due to the inhibitory activity of xeruborbactam.
[2]Aztreonam MIC corresponds to the wild-type MexAB-OprM activity but xeruborbactam MIC is low.
29 out of 30 (96.7%) strains with xeruborbactam MIC in the 8-32 µg/ml range had aztreonam alone or aztreonam with xeruborbactam (fixed 4 or 8 µg/ml) MIC in the 0.125-2 µg/ml range which is associated with a reduced activity of the MexAB-OprM efflux pump. 444 out of 461 (96.3%) strains with xeruborbactam MIC values ≥ 64 µg/ml had aztreonam MIC alone AND with xeruborbactam (fixed 8 µg/ml) ≥ 4 µg/ml which is associated with either wild-type or increased activity of the MexAB-OprM efflux pump.

Table S7. In vitro activity of beta-lactam antibiotics in combination with xeruborbactam against the strains of *K. pneumoniae*, *A. baumannii* and *P. aeruginosa* that do not produce beta-lactamases

Antibiotic	Antibiotic MIC (µg/ml) in the presence of varied xeruborbactam (µg/ml)				
	0	2	4	8	16
***Klebsiella pneumoniae* KPM1026a**					
Meropenem	0.03	0.03	0.015	0.008	NG
Tebipenem	0.03	0.03	0.016	0.004	NG
Aztreonam	0.06	0.06	0.015	0.004	NG
Ceftazadime	0.5	0.5	0.125	0.06	NG
Ceftibuten	0.06	0.03	0.015	0.008	NG
Cefepime	0.03	0.015	0.015	0.002	NG
Mecillinam	0.25	0.25	0.125	0.06	NG
***Acinetobacter baumannii* AB1007**					
Meropenem	0.5	0.125	0.06	0.03	NG
Imipenem	0.125	0.125	0.03	0.015	NG
Sulbactam	2	2	2	0.25	NG
Cefepime	2	2	1	0.25	NG
Aztreonam	32	32	16	4	NG
Pseudomonas aeruginosa* PAM1154					
Meropenem	0.5	0.125	0.06	0.06	NG
Imipenem	1	0.5	0.25	0.125	NG
Ceftazadime	1	0.25	0.25	0.25	NG
Cefepime	0.5	0.125	0.125	0.06	NG
ceftolozane	0.5	0.25	0.25	0.125	NG
Aztreonam	0.5	0.125	0.125	0.06	NG
Piperacillin	0.5	0.125	0.125	0.06	NG

* PAM1154 (*oprM::Hg*) does not have functional efflux pumps that rely on OprM, such as the major constitutively expressed efflux pump MexAB-OprM

NG, no growth, corresponds to xeruborbactam MIC of 16 µg/ml

Table S8. The potency of xeruborbactam to enhance the activity of multiple antibiotics against the KPC-3-producing clinical isolate of *K. pneumoniae* KP1074 (BAA-2814)[1]

Antibiotic	Antibiotic MIC (µg/ml) with xeruborbactam varied (µg/ml)				MIC against KPM1211[2] (µg/ml)
	none	2	4	8	
**Meropenem**	128	0.125	0.06	0.03	0.125
**Cefepime**	256	0.5	0.25	0.125	0.5
**Aztreonam**	>512	0.125	0.125	0.125	0.25
**Ceftazidime**	512	0.25	0.25	0.125	1

[1]BAA-2814 (Qpex name KP1074) contains the plasmid pKpQIL that carries bla_{KPC-3} and bla_{TEM-1}. It belongs to ST512, has a non-functional OmpK35 due to the frame-shift mutation at amino acid 42 and a partially functional OmpK36 due to the insertion of two amino acids Gly135-Asp136 which results in constricted channel.

[2]The strain KPM1211 was derived from KP1074 as a result of loss of pKpQIL. Xeruborbactam MIC for KP1074 and KPM1211 is 16-32 µg/ml.

Supplementary Figures

Figure S1. PBP band assignment in membrane preparations from various gram-negative bacteria based on the interaction with various beta-lactams.

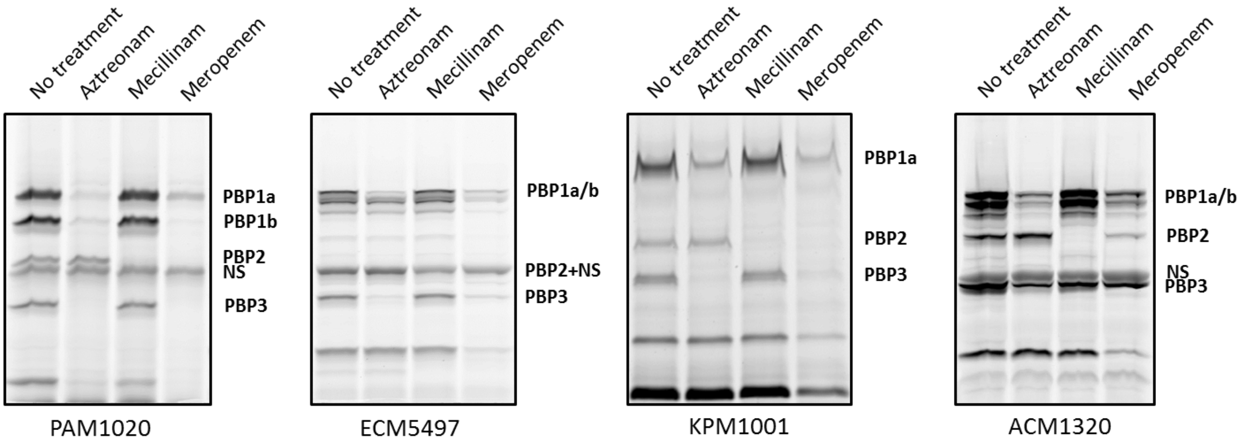

Membrane preparations from various bacterial strains were incubated with 100 µM of indicated antibiotics for 10 minutes, subsequently labeled with Boc-FL probe and subjected to gel separation and densitometry as described in Methods section. NS – nonspecifically labeled band. PAM1020 (PAO1), *P. aeruginosa*; ECM5497 (MG1655), *E. coli*; KPM1001 (ATCC 43816), *K. pneumonia*; ACM1320 (ATCC 17978), *A. baumannii*.

References

1. Nelson K, Hemarajata P, Sun D, Rubio-Aparicio D, Tsivkovski R, Yang S, Sebra R, Kasarskis A, Nguyen H, Hanson BM, Leopold S, Weinstock G, Lomovskaya O, Humphries RM. 2017. Resistance to Ceftazidime-Avibactam Is Due to Transposition of KPC in a Porin-Deficient Strain of Klebsiella pneumoniae with Increased Efflux Activity. Antimicrob Agents Chemother 61:e00989-17.
2. Lomovskaya O, Lee A, Hoshino K, Ishida H, Mistry A, Warren MS, Boyer E, Chamberland S, Lee VJ. 1999. Use of a genetic approach to evaluate the consequences of inhibition of efflux pumps in Pseudomonas aeruginosa. Antimicrob Agents Chemother 43:1340-6.
3. Lomovskaya O, Nelson K, Rubio-Aparicio D, Tsivkovski R, Sun D, Dudley MN. 2020. Impact of Intrinsic Resistance Mechanisms on Potency of QPX7728, a New Ultrabroad-Spectrum Beta-Lactamase Inhibitor of Serine and Metallo-Beta-Lactamases in *Enterobacteriaceae*, *Pseudomonas aeruginosa*, and *Acinetobacter baumannii*. Antimicrob Agents Chemother 64:e00552-20.
4. Coyne S, Courvalin P, Périchon B. 2011. Efflux-mediated antibiotic resistance in *Acinetobacter* spp. Antimicrob Agents Chemother 55:947-53.
